# A Multifunctional Nanocatalytic Metal-Organic Framework as a Ferroptosis Amplifier for Mild Hyperthermia Photothermal Therapy

**DOI:** 10.34133/research.0397

**Published:** 2024-07-01

**Authors:** Ying Deng, Duo Wang, Wenhua Zhao, Guanhua Qiu, Xiaoqi Zhu, Qin Wang, Tian Qin, Jiali Tang, Jinghang Jiang, Ningjing Lin, Lili Wei, Yichen Liu, Yuan Xie, Jie Chen, Liu Deng, Junjie Liu

**Affiliations:** ^1^ Department of Medical Ultrasound, Guangxi Medical University Cancer Hospital, Guangxi Medical University, Nanning, Guangxi, China.; ^2^ Center of Interventional Radiology and Vascular Surgery, Department of Radiology, Zhongda Hospital, Medical School, Southeast University, Nanjing, Jiangsu, China.; ^3^ Department of Oncology and Research Department, Guangxi Medical University Cancer Hospital, Guangxi Medical University, Nanning, Guangxi, China.; ^4^ Department of Hepatobiliary Surgery, Guangxi Medical University Cancer Hospital, Guangxi Medical University, Nanning, Guangxi, China.; ^5^ Hunan Provincial Key Laboratory of Micro and Nano Materials Interface Science, College of Chemistry and Chemical Engineering, Central South University, Changsha, Hunan, China.

## Abstract

Hyperthermia therapy is considered an effective anticancer strategy. However, high temperature can trigger an excessive inflammatory response, leading to tumor self-protection, immunosuppression, metastasis, and recurrence. To address this issue, we reported a multifunctional photothermal nanoplatform to achieve mild hyperthermia photothermal therapy (mild PTT) based on cisplatin (DDP) and a ferrocene metal-organic framework (MOF-Fc) nanocomposite, which can specifically enhance ferroptosis-triggered oxidative stress levels and synchronously amplify mild hyperthermia PTT-mediated anticancer responses. Both in vitro and in vivo antineoplastic results verify the superiority of mild PTT with DDP/MOF-Fc@HA. The combination of DDP and MOF-Fc exhibits Fenton catalytic activity and glutathione depletion capacity, magnifying mild hyperthermia effects via the radical oxygen species (ROS)-adenosine triphosphate (ATP)-HSP silencing pathway, with important implications for clinical hyperthermia therapy.

## Introduction

Hyperthermia therapy (HTT) has attracted considerable attention for the treatment of cancer [1–5]. However, HTT generally requires a high local temperature beyond the tolerance threshold (over 50 °C) to achieve efficient ablation of tumors; consequently, inflammation and collateral damage to normal tissues around lesions are inevitably induced by heat diffusion due to excessive hyperthermia [6–9]. Therefore, the clinical application of HTT is limited. Recently, mild PTT (below 43 °C) has emerged as an alternative to overcome this controversial issue of conventional HTT [10–12]. Unfortunately, at only 5 °C above body temperature, self-protection pathways in cancer cells can be activated to repair fever-type cell damage via the overexpression of heat shock proteins (HSPs) to increase heat tolerance [13–15]. To alleviate thermotolerance, various HSP inhibition strategies, including the use of triptolide, STA-9090, and gambogic acid [16,17], have been utilized to amplify the therapeutic efficacy of mild PTT [18,19]. However, most HSP inhibitors suffer from poor solubility, serum instability, and cytotoxicity [20,21]. Therefore, developing a therapeutic platform that simultaneously amplifies photothermal capability with HSP silencing is highly desirable for mild PTT [22–26].

Recently, the increase in membrane lipid peroxides (LPOs) and intracellular radical oxygen species (ROS) has provided a promising pathway for the cleavage of HSPs [27,28]. Moreover, ferroptosis can potentially up-regulate LPO and ROS [29–31]. Ferroptosis is susceptible to silencing HSP expression, which subsequently inhibits the thermal resistance mechanism of tumor cells [32,33]. The efficacy of the ferroptosis process intrinsically depends on the generation of high levels of ROS from the Fenton reaction. However, ROS-mediated damage to cells can be eliminated by the overexpression of glutathione (GSH), which is involved in intracellular redox balancing mechanisms [34–36]. In this regard, coordinating ferroptosis with the GSH depletion pathway may increase the vulnerability of tumors to heat [37]. However, the arrangement of the 3 mechanisms in a spatially and temporally coordinated manner is imperative. Thus, it is highly desirable to develop efficient nanoplatforms that can act as iron donors and GSH-regulating drugs and synergistically improve the efficacy of mild PTT through the alleviation of heat shock peptide (HSP)-mediated thermal resistance in tumors [38–41].

**Fig. 1.**
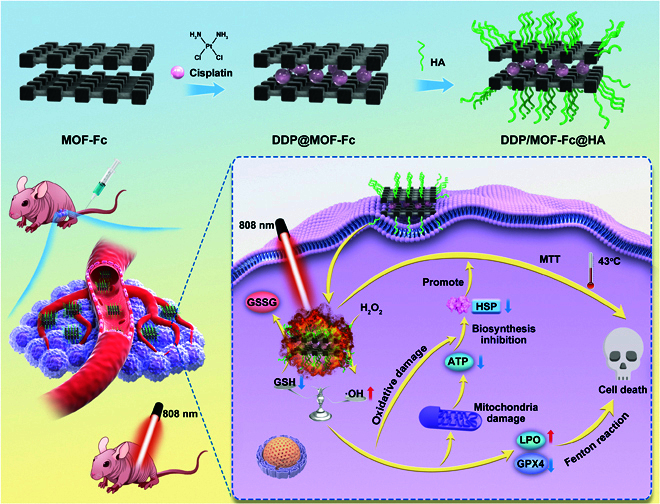
Schematic representation of the synergistic cancer therapeutic mechanism of near-infrared (NIR)-triggered DDP/MOF-Fc@HA NSs. Preparation and characterization of DDP/MOF-Fc@HA.

In this work, we designed a mild HTT with a ferroptosis/GSH-regulating system composed mainly of cisplatin (DDP) loaded with ferrocene (Fc) metal-organic frameworks (MOFs-Fc). Fc was employed to initiate ferroptosis, which not only can avoid the easy oxidation of inorganic ferrous ions during nanodrug construction or blood circulation but also exhibits inherent photothermal ability upon laser irradiation. This system also takes advantage of DDP to consume intracellular GSH to increase ROS levels. MOF-Fc exhibited a strong absorption capacity in the near-infrared region, leading to superior photothermal conversion. We extensively investigated the elimination of cancer cells through mild hyperthermia at low temperatures (38 to 43 °C) rather than at higher temperatures with this system both in vitro and in vivo. The DDP/MOF-Fc nanosheets (NSs) can efficiently catalyze ROS generation and GSH depletion to induce ferroptosis via reinforced oxidative stress. Most importantly, DDP/MOF-Fc NSs can down-regulate HSP expression by generating large amounts of ROS and decreasing adenosine triphosphate (ATP) concentrations through ferroptosis. Therefore, an important therapeutic effect was achieved by ferroptosis-improved mild PTT with DDP/MOF-Fc NSs. This study revealed a novel strategy for developing a combinational platform to synergize mild PTT and ferroptosis mechanisms, which holds great potential for the clinical improvement of cancer therapy (Fig. 1).

## Results and Discussion

### Characterization of the DDP/MOF-fc@HA nanoplatform

**Fig. 2.**
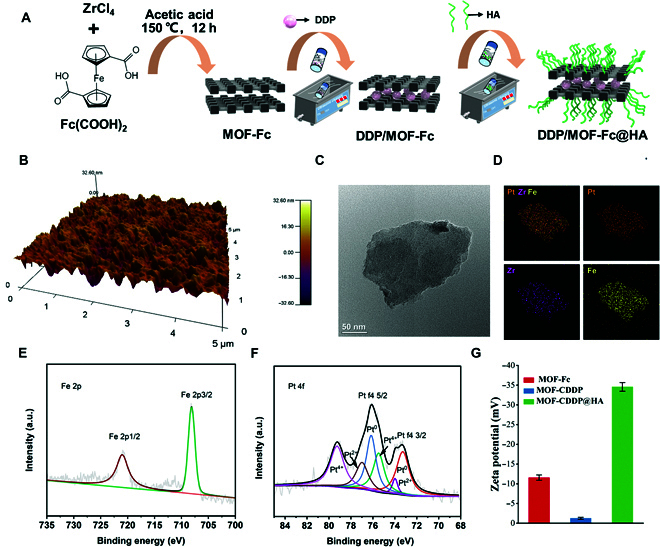
Preparation and characterization of DDP/MOF-Fc@HA. (A) Schematic drawing of the preparation procedures of DDP/MOF-Fc@HA. (B) Atomic force microscopy images of MOF-Fc. (C) High-resolution TEM images of MOF-Fc. (D) Elemental mapping images of DDP/MOF-Fc@HA. (E) X-ray photoelectron spectroscopy (XPS) of Fe 2p and (F) Pt 4f of DDP/MOF-Fc@HA. (G) Zeta potentials of MOF-Fc, DDP/MOF-Fc, and DDP/MOF-Fc@HA.

We designed a nanomedicine platform as shown in Fig. 2A. Nanosized MOF-Fc was first prepared via a one-step hydrothermal method similar to previous methods [42–47]. The morphology of the MOF-Fc was investigated via atomic force microscopy and transmission electron microscopy (TEM) (Fig. 2B and C). MOF-Fc exhibited rectangular NSs with lateral sizes of approximately 500 nm and thicknesses of approximately 30 nm. Through the porous structure of the MOF, DDP was loaded in the cavity of MOF-Fc to achieve delivery. The successful dispersion of DDP can be observed in the aberration-corrected high-angle annular dark-field scanning transmission electron microscopy image (Fig. 2D). X-ray photoelectron spectroscopy (XPS) was also conducted to confirm the successful immobilization of DDP on MOF-Fc, as shown in Fig. S1. DDP/MOF-Fc NSs contain elements such as C, O, Zr, Fe, and Pt, and fine analysis of O (1 s) and Zr (3d) diffraction peaks was performed (Figs. S2 and S3). More importantly, from the fine analysis of the Fe 2p diffraction peak in Fig. 2E, the absorption bands at 707.3 eV and 723.9 eV are ascribed to Fe 2p1/2 and Fe 2p3/2, respectively. These 2 energy bands are typical Fe^2+^ and Fe^3+^ peaks. Due to the presence of ferrous ions, the prepared MOFs can react with H_2_O_2_ to generate radical oxides through the Fenton reaction. The Pt 4f characteristic peak of DDP/MOF-Fc NSs can be fitted into 3 pairs of characteristic peaks (Fig. 2F). These pairs of peaks correspond to the 3 valence states of surface platinum atoms. The first pair of characteristic peaks is located at 71.4 eV and 75.0 eV, corresponding to the Pt 4f7/2 and Pt 4f5/2 binding energies of Pt (0) on the MOF surface, respectively. The second pair of characteristic peaks are located at 72.2 eV and 76.0 eV, corresponding to the binding energy of Pt(II). The binding energy peaks located at 74.4 eV and 77.9 eV are the third pair of characteristic peaks of Pt(IV). This result demonstrated that the DDP loaded on the MOF is mainly 0 valent, and the mutual conversion of Pt(0), Pt(II), and Pt(IV) is conducive to the adsorption and dissociation of GSH on its surface. The DDP concentration in MOF-Fc was measured to be 0.2 wt% via inductively coupled plasma–optical emission spectrometry (ICP–OES). To endow DDP/MOF-Fc with high biocompatibility, hyaluronic acid (HA) was further modified on MOF-Fc to form DDP/MOF-Fc@HA NSs. The zeta potentials of the prepared DDP/MOF-Fc@HA NSs were characterized (Fig. 2G). After the HA was coated on DDP/MOF-Fc, the surface charge changed from −1.3 mV to –34.6 mV. The x-ray diffraction pattern of the synthesized DDP/MOF-Fc@HA NSs displays a 2-dimensional structure, which is consistent with that of pristine MOF-Fc NSs (Fig. S4), indicating no substantial changes in the MOF-Fc nanostructure after modification [48]. Moreover, no Fe^2+^ leaching was detected during incubation in water or DMEM, which indicates that the DDP/MOF-Fc@HA NSs are suitable for biomedical applications.

### Photothermal activity and Fenton catalysis of the DDP/MOF-fc@HA nanoplatform

**Fig. 3.**
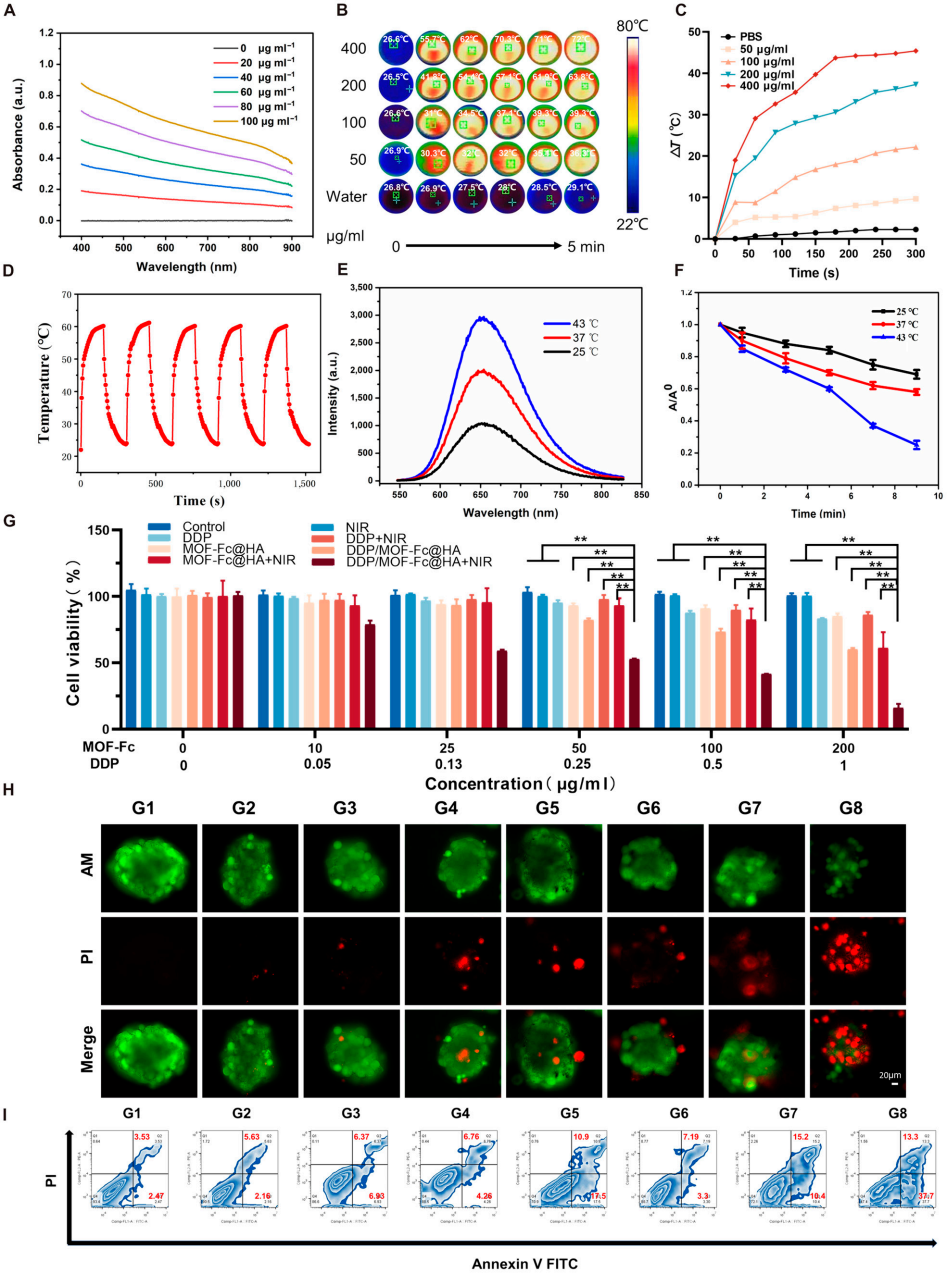
In vitro photothermal activity and Fenton catalysis. (A) UV–vis–NIR absorption spectra of MOF-Fc. (B) Infrared thermal images of different concentrations of DDP@MOF-Fc and (C) photothermal warming curves under NIR irradiation (808 nm, 1.0 W cm^−2^, approximately 10 cm from the bottom of the cell dish) for 5 min. (D) MOF-Fc was alternately heated and cooled at the same concentration for 5 cycles under irradiation with NIR (808 nm, 1.0 W cm^−2^, approximately 10 cm from the bottom of the cell dish). (E) Colorimetric reaction of TMB with DDP/MOF-Fc@HA at different temperatures. (F) Degradation of methylene blue (MB) at 654 nm by DDP/MOF-Fc@HA. (G) The cell viability profiles of Hepa 1-6 cells after incubation with different concentrations of drugs under different treatments (*n* = 4). (H) Calcein (AM)/PI costained CLSM images of 3D tumor spheres in Hepa 1-6 cells. (I) Flow cytometry patterns of Hepa 1-6 cells after Annexin V-FITC/PI costaining. For the G1 control (PBS), G2 (NIR), G3 (DDP), G4 (MOF-Fc@HA), G5 (DDP/MOF-Fc@HA), G6 (DDP+NIR), G7 (MOF-Fc@HA+NIR), and G8 (DDP/MOF-Fc@HA+NIR) groups, the cells were first incubated at the corresponding concentrations for 2 h after the NIR groups were treated by irradiation using an 808-nm near-infrared NIR (808 nm, 1.0 W cm^−2^, approximately 10 cm from the bottom of the well plate dish). **P < 0.01.

The ultraviolet–visible spectroscopy (UV–vis–NIR) absorption spectrum of MOF-Fc displays a broad absorption peak at 400 to 900 nm, which inspired us to further explore its photothermal properties (Fig. 3A). We used NIR (808 nm, 1.0 W cm^−2^, approximately 10 cm from the bottom of the cell plate) to measure the temperature change of DDP@MOF-Fc at different concentrations using a thermal imager after irradiation and obtained infrared thermal images of the corresponding nodes, as well as plotted the corresponding curve graphs. As shown in Fig. 3B, as the concentration of DDP@MOF-Fc increased, the solution temperature increased to a greater extent. For example, for DDP@MOF-Fc solutions with concentrations of 100 and 200 μg ml^−1^, the solution temperatures increased by 22.2 °C and 37.3 °C after 5 min, respectively (Fig. 3C). Temperature increases were observed in the MOF-Fc solution with increasing irradiation time, and the photothermal conversion efficiency of MOF-Fc was calculated to be approximately 49.2% [49,50]. Moreover, the temperature of MOF-Fc can be adjusted by adjusting the laser power density (Fig. S5). MOF-Fc also demonstrated excellent photothermal stability after 5 on/off cycles (Fig. 3D). Thus, MOF-Fc holds great potential as a photothermal agent for PTT in the NIR region and induces hyperthermia to enhance the subsequent catalytic capacity.

Next, we investigated the Fenton activity of DDP/MOF-Fc@HA NSs via a colorimetric reaction based on 3,3′5,5′-tetramethylbenzidine (TMB) [38,51]. Compared with DDP/MOF-Fc@HA NSs alone, a blue-colored OxTMB with maximum absorption at 652 nm was observed in the presence of H_2_O_2_ (Fig. 3E), indicating that DDP/MOF-Fc@HA NSs exhibit a catalytic capacity to generate •OH from H_2_O_2_ through the Fenton reaction. Additionally, an obvious increase in the characteristic absorption peak of OxTMB at 652 nm was measured as the temperature increased. The degradation of methylene blue (MB) at 654 nm was further studied to determine the generation capacity of the DDP/MOF-Fc@HA NSs for •OH at different temperatures (Fig. 3F). The intensity of the absorption peak decreased sharply with increasing incubation time. Compared with the other groups, the 43 °C group exhibited the greatest •OH generation efficiency. The above results indicated that the Fenton activity of DDP/MOF-Fc@HA NSs can be enhanced by photothermal properties after NIR irradiation. Moreover, the Fenton catalytic behavior of DDP/MOF-Fc@HA NSs at different temperatures followed typical Michaelis–Menten kinetics, and the enzyme kinetic parameters, including the Michaelis–Menten constant (*K*_m_) and maximum reaction rate (υ_max_), were calculated. As shown in Table S1, the υ_max_ at 43 °C is approximately 3 times that at 25 °C, suggesting that the DDP/MOF-Fc@HA NSs can function as an efficient Fenton catalyst and that the temperature can further promote the generation of •OH. To investigate the GSH binding capacity, GSH was measured with a GSH assay kit via 5,5′-dithiobis (2-nitrobenzoic acid) (DNTB) as the chromogenic reagent. After incubation with DDP/MOF-Fc@HA NSs, the depleted GSH level was calculated to be approximately 32.6%, confirming the depletion of GSH via DDP on the MOF-Fc@HA NS surface (Fig. S6). In summary, DDP/MOF-Fc can function as an efficient nanosystem for dual Fenton activity and GSH-regulating drugs.

### Therapeutic effects of DDP/MOF-fc@HA NSs in vitro

Given the excellent mild-photothermal, ROS-generated, and GSH-depletion performance of DDP/MOF-Fc@HA NSs, their therapeutic effect was further investigated at the cellular level. To facilitate the observation of DDP/MOF-Fc@HA, the IR780 was functionalized on the DDP/MOF-Fc@HA. After incubation over 2 h, an obvious red light was detected in both Hepa 1-6 and 4T1 cells, demonstrating the successful uptake of DDP/MOF-Fc@HA. Initially, due to the ability of DDP/MOF-Fc@HA NSs to endocytose tumor cells, the cellular uptake efficiency of DDP/MOF-Fc@HA NSs was explored in Hepa 1-6 cells and 4T1 cells via IR780-functionalized MOF-Fc (Fig. S7). An important red signal was observed after 2 h of incubation with DDP/MOF-Fc@HA NSs, confirming the successful internalization of DDP/MOF-Fc@HA NSs into the tumor cells. The effects of different treatments on tumor cell viability were measured using Hepa 1-6 cells and 4T1 cells via a CCK-8 assay. As the concentration of the nanoplatforms increased, the cell viability decreased (Fig. 3G and Fig. S8). Among them, the DDP/MOF-Fc@HA+NIR group exhibited the greatest cytotoxicity to Hepa 1-6 and 4T1 cells. In particular, the cell viability of the 100 μg ml^−1^ DDP/MOF-Fc@HA+NIR group was 41.6%, whereas that of the cells treated without NIR irradiation was 73.5%, indicating that the mild NIR-mediated PTT improved cell cytotoxicity. Moreover, the cytotoxicity of DDP/MOF-Fc@HA NSs toward Hepa 1-6 cells was assessed via 2D cell and 3D tumor sphere assays with calcein-AM/PI double live/dead cell staining (Fig. S9 and Fig. 3H). The DDP/MOF-Fc@HA NSs group exhibited great lethality toward both cells and spheres. Furthermore, the combined treatment of DDP/MOF-Fc@HA NSs with NIR irradiation importantly improved the ablation efficacy against cancer cells. The same trend was also observed in the 4T1 cell in vitro antitumor model (Fig. S10), which can be attributed to the synergistic effect of ferroptosis and photothermal activity resulting from DDP/MOF-Fc@HA+NIR treatment. Flow cytometry (FCM) analysis was also performed to quantify the percentage of early and late apoptotic Hepa 1-6 cells, and the maximum percentage of early and late apoptotic Hepa 1-6 cells was induced after DDP/MOF-Fc@HA+NIR treatment, with the percentage of apoptotic cells peaking at 51% (Fig. 3I and Fig. S11). A similar trend was observed in 4T1 cells after different treatments (Fig. S12). The above results demonstrated that DDP/MOF-Fc@HA+NIR can have a massive therapeutic effect on tumor cells.

### Mechanisms of DDP/MOF-fc@HA NS-mediated mild hyperthermia

**Fig. 4.**
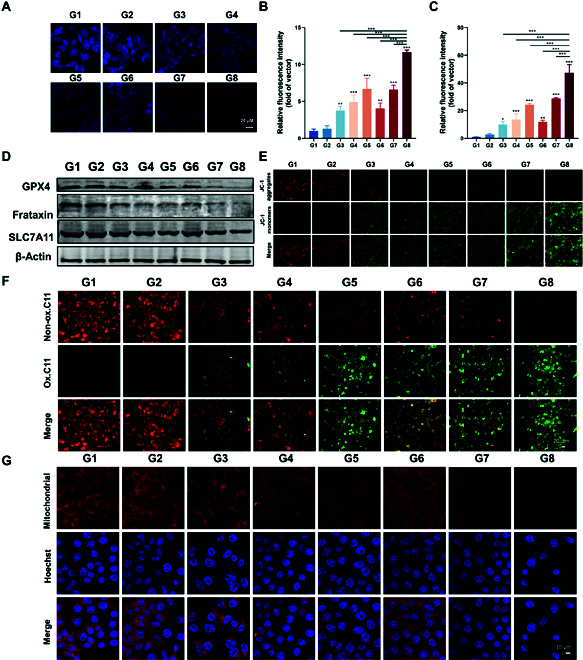
In vitro ferroptosis investigations: (A) CLSM images of Hepa 1-6 cells after treatment with a GSH indicator (i.e., ThiolTracker). (B) Corresponding quantitative fluorescence signal intensities of CLSM images of Hepa 1-6 cells after staining with an •OH indicator (i.e., the hydroxyl radical fluorescent probe O28) after different treatments. (C) Corresponding quantitative fluorescence signal intensities of CLSM images of Hepa 1-6 cells after staining with an ROS indicator (i.e., DCFH-DA) after different treatments. (D) Western blotting was used to detect changes in glutathione (GPX4), frataxin, and SLC7A11/Xct (R) protein expression in Hepa 1-6 cells. (E) CLSM images of Hepa 1-6 cells after JC-1 probe staining after different treatments were used for direct assessment of mitochondrial damage, where the expression of the JC-1 monomer (green fluorescence) was positively correlated with the extent of mitochondrial damage. (F) CLSM images of Hepa 1-6 cells after Image-iT (lipid peroxidation) and (G) mitochondrial probe staining after different treatments. G1 control (PBS); G2 (NIR); G3 (DDP); G4 (MOF-Fc@HA); G5 (DDP/MOF-Fc@HA); G6 (DDP+NIR); G7 (MOF-Fc@HA+NIR); and G8 (DDP/MOF-Fc@HA+NIR). The cells were first incubated at the corresponding concentration for 2 h after the NIR groups were treated by irradiation using an 808-nm near-infrared NIR (808 nm, 1.0 W cm^−2^, approximately 10 cm from the bottom of the well plate dish). *P < 0.05, **P < 0.01, ***P < 0.001.

Encouraged by the above performance of DDP/MOF-Fc@HA NSs, the possible mechanism of cell death was studied. Generally, GSH is overexpressed in tumor cells to compensate for redox homeostasis for tumor cell growth. Therefore, GSH depletion was initially measured with ThiolTracker as a fluorescence probe (Fig. 4A). Compared with that in the control group, there was a slight change in the fluorescence intensity in the group treated with NIR alone. However, the relative GSH level in the DDP/MOF-Fc@HA NS group decreased by 53% and 55% in the Hepa 1-6 and 4T1 cells, respectively, and the relative GSH level in the DDP/MOF-Fc@HA+NIR group decreased by 78% and 81%, respectively, in the Hepa 1-6 and 4T1 cells. The results confirmed that DDP on MOF-Fc@HA NSs can deplete intracellular GSH, and the entire process can be accelerated after NIR irradiation (Fig. S13). In addition, the intracellular •OH and ROS levels were further measured by O28 and DCFH-DA as fluorescence probes (Fig. 4B and C and Figs. S14 and S15). Compared with that in the MOF-Fc@HA NS group, a stronger fluorescence signal was observed in the DDP/MOF-Fc@HA NS group, indicating that GSH depletion by DDP can significantly facilitate the generation of ROS via the Fenton catalytic reaction. Moreover, a significant increase in fluorescence intensity was observed for both MOF-Fc@HA NSs+NIR, suggesting that a temperature increase can obviously amplify the Fenton catalytic efficiency of MOF-Fc@HA NSs. After that, we further explored the expression of key ferroptosis suppressive factors, including glutathione peroxidase (GPX4), cysteine/glutamate reverse transporter protein (SLC7A11), and frataxin, by Western blot (WB) analysis [52,53]. Compared with those of the other groups, DDP/MOF-Fc@HA+NIR irradiation obviously suppressed the expression of GPX4, SLC7A11, and frataxin (Fig. 4D), which disrupted redox homeostasis in tumor cells and subsequently induced ferroptosis. Based on ROS generation, GSH depletion, and GPX4 suppression, the intracellular LPO level was further evaluated. Image-iT (C11-BODIPY 581/591) is an oxidation-sensitive LPO-specific fluorescent probe capable of accumulating in cellular membranes. Upon oxidation of C11-BODIPY 581/591, the maximum emission peak shifts from 590 nm (red) to 510 nm (green) and remains intrinsically lipophilic, which is favorable for membrane LPO detection. Confocal laser scanning microscope (CLSM) images revealed that in the DDP/MOF-Fc@HA and MOF-Fc@HA+NIR treatment groups, the attenuation of red fluorescence and enhancement of green fluorescence changed to a certain extent. Moreover, a very weak red fluorescence signal and bright green fluorescence were observed in the DDP/MOF-Fc@HA+NIR treatment groups (Fig. 4F). The same trend was also observed in the 4T1 cells (Fig. S16). Then, the membrane-permeant fluorescent dye 5,5′,6,6′-tetrachloro-1,1′,3,3′-tetraethylimidacarbocyanine (JC-1) was used to monitor the change in the mitochondrial membrane potential (MMP), which exhibited green fluorescence while remaining as a J-monomer in the damaged mitochondria (Fig. 4E) [54]. For the control and NIR groups, the G/R ratios were approximately 0.04 and 0.05, respectively. After incubation with MOF-Fc@HA NSs and DDP/MOF-Fc@HA NSs, the G/R values increased to 0.65 and 1.33, respectively. Furthermore, the G/R value of DDP/MOF-Fc@HA+NIR increased to 8.4 (Fig. S17). Additionally, direct mitochondrial staining of Hepa 1-6 hepatocellular carcinoma cells was performed, and the red fluorescence signal was negatively correlated with mitochondrial damage (Fig. 4G and Fig. S18). The Hepa 1-6 cells treated with DDP/MOF-Fc@HA+NIR exhibited more intense red fluorescence than did the other groups. Bio-TEM revealed mitochondria in the Hepa 1-6 cells treated with DDP/MOF-Fc@HA+NIR (Fig. S19). As shown in Fig. 4G, DDP/MOF-Fc@HA NSs caused an obvious decrease in volume, an increase in the mitochondrial membrane density, the disappearance of mitochondrial cristae, and a swelling phenotype, which are typical features of mitochondrial functional impairment through ferroptosis. The above results clearly verified that DDP/MOF-Fc@HA NSs can induce ferroptosis in tumor cells and that NIR irradiation can further improve the efficiency of ferroptosis.

**Fig. 5.**
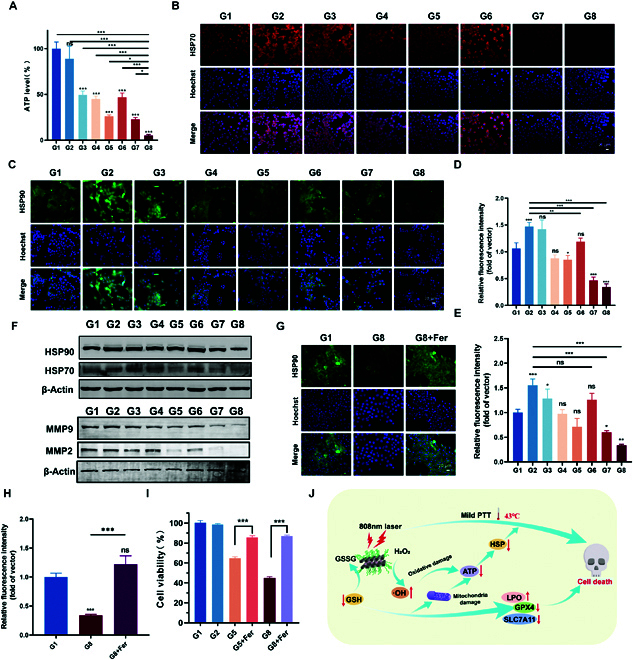
Modulatory effect of mild hyperthermia via ferroptosis. (A) ATP concentration in Hepa 1-6 cells after different treatments (*n* = 4). (B) CLSM images of Hepa 1-6 cells after HSP70 immunofluorescence staining and (D) the corresponding quantitative fluorescence signal intensities. (C) CLSM images of Hepa 1-6 cells after HSP90 immunofluorescence staining and (E) the corresponding quantitative fluorescence signal intensities. (F) WB analysis of HSP70, HSP90, MMP2, and MMP9 protein levels in Hepa 1-6 cells subjected to different treatments. (G) CLSM images of Hepa 1-6 cells after HSP90 immunofluorescence staining after the introduction of ferrostatin-1 (Fer) and (H) the corresponding quantitative fluorescence signal intensities. (I) The cell viability profiles of Hepa 1-6 cells after the introduction of ferrostatin-1 (Fer) (*n* = 4). (J) Schematic illustration of the enhancement of iron-induced apoptosis by mild PTT. For the G1 control (PBS), G2 (NIR), G3 (DDP), G4 (MOF-Fc@HA), G5 (DDP/MOF-Fc@HA), G6 (DDP+NIR), G7 (MOF-Fc@HA+NIR), and G8 (DDP/MOF-Fc@HA+NIR) groups, the cells were first incubated at the corresponding concentrations for 2 h after the NIR groups were treated by irradiation using an 808-nm near-infrared NIR (808 nm, 1.0 W cm^−2^, approximately 10 cm from the bottom of the well plate dish). *P < 0.05, **P < 0.01, ***P < 0.001.

Furthermore, we studied the modulatory effect of ferroptosis on mild hyperthermia. We speculated that the increase in oxidative stress in tumor cells can inhibit ATP generation [55,56] and subsequently down-regulate heat shock proteins (HSPs), which are protective factors in cells under high-temperature stress conditions [57]. To confirm this hypothesis, the intracellular ATP levels were evaluated with an ATP assay kit. As shown in Fig. 5A, NIR alone reduced the ATP content by approximately 10%, DDP/MOF-Fc@HA NS treatment reduced the ATP content by approximately 73.6%, and DDP/MOF-Fc@HA+NIR treatment further reduced the ATP content by approximately 14.3%. This is consistent with the trend of ROS levels, a phenomenon that further demonstrates DDP@MOF-Fc+NIR’s ability to amplify oxidative stress, effectively blocking the production of ATP and the synthesis of HSPs. The levels of the ATP-dependent proteins HSP70 and HPS90 were further investigated via immunofluorescence experiments (Fig. 5B and C). Compared with those in cells treated with NIR irradiation, HSP70 and HSP90 were clearly down-regulated in cells treated with DDP/MOF-Fc@HA NSs, indicating that efficient ferroptosis can silence HSP generation. As shown in Fig. 5D and E, NIR alone improved the HSP70 and HSP90 contents by approximately 147.2% and 155.5%, respectively. However, the MOF-Fc@HA+NIR treatment reduced the HSP70 and HSP90 contents by approximately 46.7% and 60.2%, respectively, indicating that ferroptosis can boost mild PTT. In particular, the expression levels of HSP70 and HSP90 were much lower in the cells treated with DDP/MOF-Fc@HA+NIR, which were reduced by approximately 34.2% and 34%, respectively, confirming that mild PTT can be further enhanced significantly by improving ferroptosis via GSH depletion. Western blotting was also conducted to measure the levels of HSP70 and HPS90 in Hepa 1-6 cells (Fig. 5F), and the results were consistent with the CLSM results. Studies have shown that tumor invasiveness is importantly correlated with increased MMP expression, and it is generally believed that inhibiting the activity of MMP2 and MMP9 can meaningfully inhibit tumor invasion and metastasis. WB analysis revealed that after treatment with DDP/MOF-FC@HA+NIR, the expression of MMP2 and MMP9 was significantly inhibited, which could inhibit tumor metastasis (Fig. 5F). To verify this phenomenon, restored HSP90 expression was investigated via the introduction of ferrostatin-1 (Fer), a ferroptosis inhibitor, into the DDP/MOF-Fc@HA+NIR group (Fig. 5G and H). Interestingly, the expression level of HSP90 in the DDP/MOF-Fc@HA+NIR treatment group was significantly greater than the original level in the control group after the introduction of Fer into the Hepa 1-6 cells. This result verified that HSP expression can be efficiently suppressed by ferroptosis via ATP depletion and high ROS generation, which provided a pathway to augment the mild PTT efficacy of the improved ferroptosis pathway. Subsequently, the CCK-8 assay was conducted to verify the enhanced therapeutic effect of ferroptosis on mild PTT.

A CCK assay was performed to examine the therapeutic effect of ferroptosis promotion on mild PTT. As shown in Fig. 5I and Fig. S20, a significant therapeutic effect was observed in the DDP/MOF-Fc@HA+NIR group compared to the DDP/MOF-Fc@HA group due to the improvement of mild PTT by ferroptosis. However, in the DDP@MOF-Fc+NIR+Fer group, the cell viability rate increased once ferroptosis was inhibited, confirming that the superior therapeutic effect of DDP/MOF-Fc@HA+NIR is mainly due to ferroptosis-reinforced mild NIR PTT instead of mild NIR-PTT boosting ferroptosis (Fig. 5J).

### In vivo antitumor evaluation and mechanism analysis

**Fig. 6.**
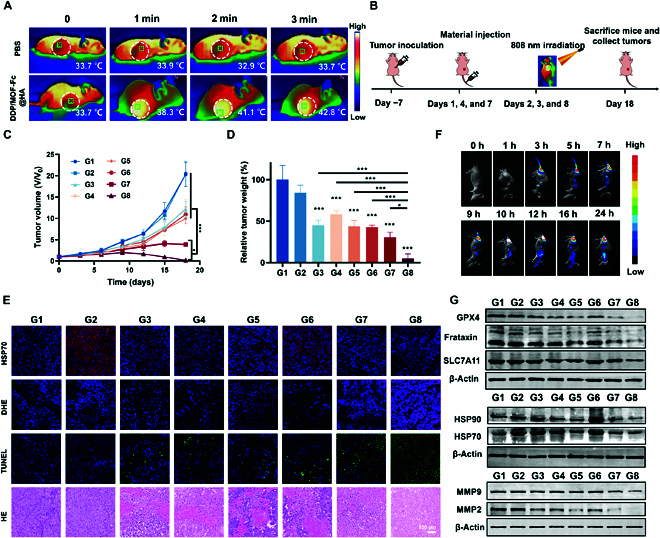
In vivo antitumor effects and mechanism evaluation. (A) Mouse tumor warming thermogram after 3 min (808 nm, 1.5 W cm^−2^). (B) The in vivo treatment schedule. (C) Time-dependent relative tumor volume curves and (D) relative tumor weights for each group of mice after different treatments (*n* = 4). (E) At the end of the experimental period, H&E light microscopy images of tumor sections isolated from mice bearing Hepa 1-6 tumors in separate groups (G1 to G8) and CLSM images of deoxynucleotidyl transferase-mediated nick end labeling, DHE, and HSP70 immunofluorescence staining. (F) Time-dependent in vivo fluorescence images of mice bearing Hepa 1-6 hepatocellular carcinomas via the tail vein after IR780/DDP/MOF-Fc@HA treatment. (G) At the end of the experimental period, tumors isolated from mice bearing Hepa 1-6 cells in separate groups (G1 to G8) were collected, and WB analysis of GPX4, SLC7A11, frataxin, HSP70, HSP90, MMP2, and MMP9 protein levels was performed. G1, control (PBS); G2, NIR; G3, DDP; G4, MOF-Fc@HA; G5, DDP/MOF-Fc@HA; G6, DDP+NIR; G7, MOF-Fc@HA+NIR; and G8, DDP/MOF-Fc@HA+NIR. On day 1, day 4, and day 7, the mice were injected with drugs (for nanoparticles, DDP/MOF-Fc@HA dose: 1.0 mg (kg BW)^−1^, DDP dose: 1.0 mg (kg BW)^−1^; for free drug, MOF-Fc@HA dose: 1.0 mg (kg BW)^−1^, DDP dose: 1 mg (kg BW)^−1^) into the tail vein and subjected to NIR irradiation (808 nm, 1.5 W cm^−2^, approximately 10 cm from the tumor surface) for 3 min. *P < 0.05, ***P < 0.001.

Inspired by the superior performance of DDP/MOF-Fc@HA+NIR treatment, in vivo antitumor studies were subsequently performed. First, to evaluate the effect of NIR-triggered mild PTT, saline or DDP/MOF-Fc@HA NSs were injected into the tail vein on the previous day, and the mice were anesthetized and irradiated with NIR (808 nm, 1.5 W cm^−2^, approximately 10 cm from the tumor surface) before irradiation with NIR. The tumor heating curves of the mice were recorded with a thermographic camera, and the corresponding elevation curves were plotted. DDP/MOF-Fc@HA was heated to 9.1 °C after irradiation with NIR (808 nm, 1.5 W cm^−2^, approximately 10 cm from the tumor surface) for 3 min, and the results were compared with those of no obvious warming after saline injection, showing the effect of mild thermotherapy (Fig. 6A and Fig. S21). As shown in the schematic diagram (Fig. 6B), 4-week-old male nude mice were subjected to tail vein injection on days 1, 4, and 7 and NIR (808 nm, approximately 10 cm from the tumor surface) irradiation on days 2, 5, and 8. At the end of the experiment on day 18, the tumors were collected from the groups, and their relative tumor growth curves were obtained (Fig. 6C). The relative weights of the tumors after isolation showed that the DDP/MOF-Fc@HA+NIR-treated tumors had the greatest suppressive effect on the tumors (Fig. 6D). This can be attributed to the triple combination of ROS generation, GSH depletion and mild hyperthermia, which increased the heat resistance of mild PTT. During the experimental period, no fluctuation in the body weight of the hormonal mice in each group was observed (Fig. S22), which ensured the safety of the treatment. No obvious changes were detected in normal tissues or blood, no obvious hemolysis was detected after DDP/MOF-Fc@HA+NIR treatment, and the H&E-stained structures of the heart, liver, spleen, lung, and kidney were normal between the treatment groups (Figs. S23 to S25). To further investigate the antitumor mechanism, the isolated tumors were first examined pathologically. H&E staining showed that the greatest reductions in nuclei and cell density were observed in the DDP/MOF-Fc@HA+NIR group. Similarly, the rate of deoxynucleotidyl transferase-mediated nick end labeling apoptosis was the lowest in the DDP/MOF-Fc@HA+NIR group. In addition to the acquired dihydroethidium (DHE) immunofluorescence images used to examine the extent of ROS damage, a prominent level of fluorescence was also observed after treatment in the DDP@MOF-Fc+NIR group (Fig. 6E). While HSPs are ATP chaperone proteins and the synthesis, expression, and function of HSPs cannot be dissociated from intracellular ATP, DDP/MOF-Fc@HA+NIR treatment resulted in the greatest accumulation of ROS and increased silencing of HSPs to the extent that their antitumor efficacy was greatly increased. In addition, time-dependent in vivo fluorescence image analysis of mice bearing Hepa 1-6 hepatocellular carcinoma after tail vein injection of IR780/DDP/MOF-Fc@HA NSs and ex vivo organ and tumor fluorescence image analysis after 24 h showed that DDP/MOF-Fc@HA NSs have good targeting ability (Fig. 6F and Fig. S26). We performed immunoblot analysis on isolated tumor tissues to further validate the changes in the expression of key cellular proteins, and after DDP/MOF-Fc@HA+NIR treatment, iron death pathway-related proteins such as GPX4, SLC7A11, frataxin, MMP2, and MMP9 were importantly reduced (Fig. 6G). In conclusion, this suggested that DDP/MOF-Fc@HA+NIR treatment was also successful and effective in increasing iron death levels in vivo. Next, by analyzing the expression of heat shock proteins in vivo, it was evident that HSP70 and HSP90 were successfully suppressed by DDP/MOF-Fc@HA+NIR. This finding is consistent with the results of DHE staining for detecting ROS levels in tumors. Overall, DDP/MOF-Fc@HA NSs exhibited superior efficiency in inhibiting tumor growth with compatibility. This strategy provides new opportunities to exploit ferroptosis to enhance mild PTT rather than employing HTT, which has severe toxic side effects.

## Conclusion

In summary, we successfully constructed a nanoparticle that coordinates the 3 mechanisms of photothermal treatment, ferroptosis, and GSH depletion by loading DDP onto the surface of MOF-Fc NSs. Ferrocene was shown to catalyze the conversion of H_2_O_2_ to cytotoxic •OH in the highly H_2_O_2_ acidic microenvironment of tumors, as well as the Fenton reaction between the Fe^2+^ therein and the high hydrogen peroxide (H_2_O_2_) in the tumor microenvironment, which induces cancer cells to undergo iron death. DDP binds directly to GSH to decrease the GSH level, which leads to the inactivation of GSH and GPX4, promoting iron death. The combination of the two further successfully promotes substantial ROS accumulation, reduces the expression of heat shock proteins, including mitochondrial membrane depletion, and inhibits energy metabolism represented by ATP depletion, thereby enhancing the effects of mild photothermal therapy. Taken together, our proposed strategy validates the concept of boosted ferroptosis-improved mild PTT based on the coordination of 3 mechanisms to overcome the heat resistance limitation of conventional photothermal therapies, and this therapeutic mechanism is expected to be an effective tool for enhancing the efficacy of mild thermotherapy.

## Materials and Methods

### Cell lines and animals

A mouse breast cancer cell line (4T1) and mouse liver cancer cell line (Hepa 1-6) were purchased from the American Type Culture Collection (ATCC) (Virginia, USA). The cells were cultured in a humidified incubator at 37 °C and 5% CO_2_. Nude mice were purchased and managed by Guangxi Experimental Animal Center. The ethics committee of Guangxi Medical University Cancer Hospital approved all the animal experiments.

### Preparation of DDP/MOF-fc@HA NSs

Zr-Fc MOF was synthesized according to previous solvent thermal procedures with modifications. Briefly, glacial acetic acid (75 mmol), ZrCl_4_ (1.5 mmol), and Fc(COOH)_2_ (1.5 mmol) were mixed with DMF (45 ml) and ultrasonicated for 10 min. After the hydrothermal reaction, the resulting mixture was transferred to a Teflon container (100 ml) for 12 h. The as-obtained MOF-Fc was dispersed in a DDP (100 μg ml^−1^) aqueous solution and sonicated in an ultrasonic bath (100 W, 20 kHz) for 30 min. The obtained DDP-modified MOF-Fc was collected through centrifugation, washed twice with water, dispersed in water, stored at 4 °C for further use, and named DDP/MOF-Fc. Finally, the prepared DDP/MOF-Fc was further mixed with HA (1 mg ml^−1^) in an ultrasonic bath (100 W, 20 kHz) for 30 min. The obtained HA-modified DDP/MOF-Fc was collected through centrifugation, washed twice with water, dispersed in water, and stored at 4 °C for further use and named DDP/MOF-Fc@HA NSs.

### In vitro cytotoxicity of DDP/MOF-fc@HA NSs

Live/dead fluorescent imaging of treated cells was achieved by calcein-AM and propidium iodide (PI) staining of Hepa 1-6 and 4T1 cells. The cells per well were inoculated in 24-well plates and divided into 8 groups as described above. The cells were first incubated at the corresponding concentrations for 2 h (doses: DDP/MOF-Fc@HA: 100 μg ml^−1^, DDP: 0.5 μg ml^−1^, and MOF-Fc@HA: 100 μg ml^−1^), after which the cells were subjected to the abovementioned treatments. After 24 h of continuous incubation, the medium was removed, and the cells were washed twice with phosphate buffer saline (PBS). Then, 1,000 μl of calcein (AM)/PI was added to the cells at a ratio of 1:1,000, and the cells were incubated for 15 min at 37 °C in the dark in a 5% CO_2_ incubator. Cytoplasmic green fluorescence representing live cells was recorded at λ_ex_ = 494 nm and λ_em_ = 517 nm, and red fluorescence representing dead cells was captured at λ_ex_ = 535 nm and λ_em_ = 617 nm.

For immunofluorescence staining of 3D multicellular tumor spheroids, Hepa 1-6 cells in the logarithmic growth phase were suspended in serum-free medium supplemented with the growth factors EGF and FGF-2 and then inoculated in ultralow adsorption surface 96-well plates at a density of 1 × 10^5^ cells/well. Subsequently, the plates were incubated in a humidified incubator at 37 °C with 5% CO_2,_ and half of the fluid was replaced during incubation to form 3D multicellular tumor spheroids after 15 days. The spheroids were divided into 8 groups as described above. Calcein (AM)/PI was added to the wells with medium at a ratio of 1:1,000, and the plates were incubated for 15 min in the dark at 37 °C in a 5% CO_2_ incubator and washed twice with PBS before observation. The images were observed and recorded on a confocal microscope (Zeiss, LSM710e, Oberkochen, Germany).

For FCM analysis, Hepa 1-6 and 4T1 cells were washed twice with PBS, and the cells and supernatant were collected and resuscitated with 400 μl of PBS. The cells were incubated with 5 μl of Annexin V-fluorescein isothiocyanate (FITC) and 10 μl of PI for 20 min at 37 °C and 5% CO_2_ in a cell culture incubator, and the data were analyzed with a CytoFlex S flow cytometer (Beckman Coulter, China) and FlowJo software (version 10.5.3, TreeStar).

### In vitro ferroptosis investigations

To measure the changes in the MMP, the JC-1 fluorescent probe (diluted at 1:200) was added to the Hepa 1-6 and 4T1 cells, which were then incubated for 20 min in a humidified incubator at 37 °C under 5% CO_2_ and measured by fluorescence microscopy. To measure the changes in mitochondrial distribution, the nuclei of Hepa 1-6 and 4T1 cells were first stained with Hoechst 33342 for 15 min. After being washed twice with PBS, the cells were stained with M16, a fluorescent probe for mitochondrial staining (diluted at 1:200), and incubated in a humidified incubator at 37 °C and 5% CO_2_ for 20 min. To measure the changes in lipid peroxidation, the cells were fixed with 4% paraformaldehyde and blocked with Hoechst 33342 for 15 min. Lipid peroxidation was assessed using an Image-iT Lipid Peroxidation Kit, and the samples were stained with a dye at a final concentration of 10 μM for 30 min. These cells were observed via confocal microscopy (Zeiss, LSM710e, Oberkochen, Germany).

At high MMPs, JC-1 aggregates in the mitochondrial matrix to form polymers (J-aggregates), and red fluorescence is produced, which is detected at an excitation wavelength of 585 nm and an emission wavelength of 590 nm.

### In vitro immunofluorescence analysis and ATP levels

To measure the changes in cellular HSP70 and HSP90 expression, Hepa 1-6 cells were fixed with 4% (w/v) PBS-buffered paraformaldehyde for 15 min, permeabilized with 0.5% (v/v) Triton X-100 for 5 min at room temperature, and sequentially blocked with 5% FBS for 15 min. The cells were washed with PBS and incubated with an antibody according to the manufacturer’s instructions at 4 °C. The cells were incubated with primary antibodies overnight. The primary antibodies used were HSP70 (diluted at 1:100) and HSP90 (diluted at 1:200). After washing twice with PBS, the cells were incubated with Cy3-labeled goat anti-rabbit IgG H & L or Alexa Fluor 488-labeled goat anti-rabbit IgG H & L for 1 h at room temperature, after which the nuclei were stained with Hoechst 33342. The cells were observed via confocal microscopy (Zeiss, LSM710e, Oberkochen, Germany).

To measure the intracellular ATP levels, Hepa 1-6 cells were cultured in 6-well plates with 200 μl of ATP lysate/well. After centrifugation at 12,000 *g* at 4 °C for 5 min, the intracellular ATP levels in the obtained supernatants were measured with an ATP test kit. First, 100 μl of ATP assay working solution was added to the test wells of a dedicated covered-well plate (BS-MP 96 B, Biosharp, China) and left at room temperature for 3 to 5 min to allow all background ATP to be consumed. Second, 20 μl of the sample or ATP standard solution was added to the test wells and immediately assayed by a photometer (Thermo, Waltham, USA). In addition, an absorption–concentration standard curve was prepared to calculate the ATP concentration of the sample.

### Fer addition-induced cytotoxicity assay

To test the cytotoxicity of the added Ferritin-1, Hepa 1-6 and 4T1 cells (3 × 10^3^ cells/well) were spread in 96-well plates, Fer reagent was added to the Hepa 1-6 and 4T1 cells at a final concentration of 10 μM for 24 h, and the drug medium (DDP/MOF-Fc@HA: 100 μg ml^−1^) was replaced for 24 h. The OD values were measured at 450 nm, and cell viability was analyzed by a CCK-8 assay.

### Animals and tumor model

Male nude mice (4 weeks old) were purchased from and managed by the Guangxi Experimental Animal Center. The ethics committee of Guangxi Medical University Cancer Hospital approved all the animal experiments. Hepa 1-6 subcutaneous tumor models were generated by injecting 100 μl of a Hepa 1-6 cell suspension (1 × 10^7^ cells/ml). The tumor volume (*V*) was calculated as follows: *V* = (*a* × *b*^2^)/2, where a and b are the length and width of the tumor, respectively.

### Animal models and antitumor therapy

The tumor-bearing mice (size ~ 50 mm^3^) were randomly classified into 8 groups (*n* = 4): G1 Control (PBS); G2 (NIR); G3 (DDP); G4 (MOF-Fc@HA); G5 (DDP/MOF-Fc@HA); G6 (DDP+NIR); G7 (MOF-Fc@HA+NIR); and G8 (DDP/MOF-Fc@HA+NIR). On day 1, day 4, and day 7, the mice were injected with drugs [for nanoparticles, DDP/MOF-Fc@HA dose: 1.0 mg (kg BW)^−1^, DDP dose: 1.0 mg (kg BW)^−1^; for the free drug, MOF-Fc@HA dose: 1.0 mg (kg BW)^−1^, DDP dose: 1 mg (kg BW)^−1^] into the tail vein and subjected to NIR irradiation (808 nm, 1.5 W cm^−2^, approximately 10 cm from the tumor surface) for 3 min. Tumor volume and mouse weight were recorded every 3 days. Mice were sacrificed after 18 days of treatment, and subcutaneous tumor tissues were collected for immunofluorescence staining, while heart, liver, spleen, lung, and kidney tissues were collected for H&E staining.

### In vivo thermal efficacy

Nude mice with subcutaneous implantation of tumors on the right back (tumor volume approximately 150 mm^3^) were anesthetized, and the mice were divided into the PBS group and the DDP/MOF-Fc@HA group and irradiated with a near-infrared laser (808 nm, 1.5 W cm^−2^, approximately 10 cm away from the tumor surface). The ability of DDP/MOF-Fc@HA to produce thermotherapy was monitored. The tumors were irradiated separately (1, 2, and 3 min). Thermal images and warming curves were recorded with a thermal imaging camera.

### In vivo fluorescence imaging of live animals

After administration for 0 to 24 h, the nude mice were sacrificed after 24 h, and the major organs were removed to observe the biodistribution of the nanoparticles in the tumors, heart, liver, spleen, lungs, and kidneys.

### Statistical analysis

All experiments were performed at least 3 times. The data were analyzed by GraphPad Prism 8.3 software and are presented as the mean ± standard deviation (SD). Unpaired Student,s *t* test and one-way analysis of variance (ANOVA) were used to compare 2 groups and multiple groups, respectively. *P < 0.05, **P < 0.01, and ***P < 0.001 indicated statistical significance.

## Data Availability

The data that support the findings of this study are available from the corresponding authors upon reasonable request.

## Supplementary Materials

Supplementary 1 Supplementary Materials and Methods Figs. S1 to S26 Table S1 Supporting Information original data
