# Supplementary material for: A Multifunctional Nanocatalytic Metal-Organic Framework as a Ferroptosis Amplifier for Mild Hyperthermia Photothermal Therapy
Source: Research (Wash D C). 2024 Jul 1;7:0397. doi: 10.34133/research.0397 (PMC11214948; doi:10.34133/research.0397)
Supplement: Supplementary 1 — Supplementary Materials and Methods Figs. S1 to S26 Table S1 Supporting Information original data [file research.0397.f1.zip › Supporting Information with highlted in blue.docx]

Supplementary Materials and Methods

A Multifunctional Nanocatalytic Metal-Organic Framework as a Ferroptosis Amplifier for Mild Hyperthermia Photothermal Therapy

Ying Deng^1^†, Duo Wang^1,5^†^*^, Wenhua Zhao^2^†, Guanhua Qiu^1^, Xiaoqi Zhu^1^, Qin Wang^1^, Tian Qin^1^, Jiali Tang^1^, Jinghang Jiang^1^, Ningjing Lin^1^, Lili Wei^1^, Yichen Liu^1^, Yuan Xie^2^, Jie Chen^3^, Liu Deng^4*^, Junjie Liu^1*^

Ying Deng, Duo Wang^*^, Wenhua Zhao, Guanhua Qiu, Xiaoqi Zhu, Qin Wang, Tian Qin, Jiali Tang, Jinghang Jiang, Ningjing Lin, Lili Wei, Yichen Liu, Yuan Xie, Jie Chen, Junjie Liu^*^

^1^Department of Medical Ultrasound, ^2^Department of Oncology and Research Department, ^3^Department of Hepatobiliary Surgery, Guangxi Medical University Cancer Hospital, Guangxi Medical University. China.

*Address correspondence to: Duo Wang; [wangduo2022@126.com](mailto:wangduo2022@126.com) and Junjie Liu; [liujunjie@gxmu.edu.cn](mailto:liujunjie@gxmu.edu.cn)

Liu Deng^*^

^4^Hunan Provincial Key Laboratory of Micro and Nano Materials Interface Science, College of Chemistry and Chemical Engineering, Central South University, China.

*Address correspondence to: [dengliu@csu.edu.cn](mailto:dengliu@csu.edu.cn)

Duo Wang^*^

^5^Center of Interventional Radiology & Vascular Surgery, Department of Radiology, Zhongda Hospital, Medical School, Southeast University, China

†These authors contributed equally to this work.

Part A: Experimental Section

Materials and Characterizations

Zirconium tetrachloride (ZrCl_4_, ≥99.9% metal basis), 1,1′-ferrocenedicarboxylic acid [Fc(COOH)_2_, >98%], glacial acetic acid (CH_3_COOH, AR), methylene blue trihydrate [MB, ≥90% (HPLC)], and hydrogen peroxide [H_2_O_2_, 30%] were obtained from Aladdin Co., Ltd (Shanghai, China). Dimethylformamide (DMF, AR), 3,3′,5,5′-tetramethylbenzidine (TMB) and Glutathione Assay Kit were purchased from Sigma-Aldrich Co., Ltd (Missouri, USA). Cisplatin (for injection, lyophilised) was purchased from Qilu Pharmaceutical Co., Ltd (Jinan, China). IR780 iodide (IR780) (≥95% content) was purchased from Sigma Aldrich Trading Co., Ltd (Shanghai, China). HA (Hyaluronic acid) was purchased from Macklin Co., Ltd (Shanghai, China). Image-iT^TM^ (lipid peroxidation) and ThiolTracker™ Violet (glutathione assay) were purchased from ThermoFisher Scientific Co., Ltd (Waltham, USA). Adenosine triphosphate (ATP) Assay Kit, Hoechst 33342, DCFH-DA Fluorescent Probe, Cy3-labelled goat Anti-Rabbit IgG H & L, Alexa Fluor 488-labelled goat Anti-Rabbit IgG H & L, Phosphatase Inhibitor Mixture, Bicinchoninic Acid (BCA) Protein Assay Kit, WB Transfer Buffer, TritonX-100, and QuickBlock™ blocking buffer were purchased from Beyotime Biotechnology Inc (Shanghai, China). NP40 lysis buffer was purchased from Biosharp Co., Ltd (Anhui, China). Annexin V-FITC apoptosis detection kit, M16 mitochondrial staining fluorescent probe, and hydroxyl radical fluorescent probe were purchased from BestBio Co., Ltd (Shanghai, China). GPX4, SLC7A11, HSP70, HSP90,MMP9 Rabbit pAb antibodies were purchased from Abconal Co., Ltd (Wuhan, China). β-actin and MMP2 Rabbit pAb antibodies were purchased from Proteintech Co., Ltd (Wuhan, China). Frataxin and goat anti-rabbit IgG H & L (Alexa Fluor® 680) were purchased from Abcam Co., Ltd (Cambridge, England). Ferrostatin-1 (Fer) was purchased from MCE (New Jersey, USA). DMEM medium, 0.25% trypsin-EDTA, Dihydrochloride (DAPI), and penicillin/streptomycin (P/S) were purchased from Solarbio Science & Technology Co., Ltd (Beijing, China). Fetal bovine serum (FBS) was obtained from Wisent Biotechnology Co., Ltd (Nanjing, China). CCK8 assay kit was obtained from Dojindo Laboratories Co. Ltd (Shanghai, China). PAGE gel rapid preparation kit (10%) and PAGE gel rapid preparation kit (12.5%) were purchased from Epizyme Biomedical Technology Co., Ltd (Shanghai, China). Cell culture vessels were purchased from Corning Co., Ltd (Corning, NY, USA). The handheld thermography thermal imaging camera was purchased from HIKVISION Co., Ltd (Hangzhou, China).

The morphology of the prepared nanoplatform was observed on the transmission electron microscope (TEM, Titan G260-300, FEI, USA) and atom force microscope (AFM, MFP-3D-S, Asylum research, USA) images. The UV-vis absorption spectra were measured by a UV-2450 spectrophotometer (Shimadzu, Japan). The size and zeta potential of the material were measured by the dynamic light scattering (DLS) on a Malvern Zetasizer Nano ZS instrument (Malvern, UK). The powder X-ray diffraction data were collected on X-pert Powder diffractometers using Cu Kα radiation sources (λ = 1.54178 Å, and 2θ = 0–40°), and X-ray photoelectron spectroscopy (XPS) (Thermo scalable, USA), model 250XI. The fluorescence images were observed on confocal microscope (Zeiss, LSM710e, Oberkochen, [Germany](https://fanyi.so.com/?src=onebox#Germany)) and confocal laser scanning microscopy (CLSM) (Zeiss, LSM980, Oberkochen, [Germany](https://fanyi.so.com/?src=onebox#Germany)).

**Inductively coupled plasma‒optical emission spectrometry (ICP‒OES)**

The content of DDP in the nanostructures was determined by ICPOES730 (Agilent, USA) analysis to clarify the drug loading.

Cell culture

Mouse breast cancer cell line (4T1) and mouse liver cancer cell line (Hepa 1-6) were cultured in a humidified incubator at 37°C, 5% CO_2_. To assess cellular uptake of IR780/DDP/MOF-Fc@HA, the DDP/MOF-Fc@HA NSs were modified with dye IR780. Hepa 1-6 and 4T1 cells were inoculated into 24-well plates on 14 mm cell crawlers respectively, at a density of 1.0 × 10^5^ cells/well. The original medium was replaced with 500 μL of medium containing concentration (100 μg mL^-1^) of IR780/DDP/MOF-Fc@HA and incubated for 0 hour to 2 hours. After washing twice with PBS, the cells were fixed with 4% paraformaldehyde and blocked with DAPI and passed through a confocal laser scanning microscopy (CLSM) (Zeiss, LSM980, Oberkochen, [Germany](https://fanyi.so.com/?src=onebox#Germany)).

*In vitro* Western blot analysis

Western blot method was used to detect the changes of glutathione GPX4, Frataxin, SLC7A11/Xct (R), HSP70, and HSP90 protein expression in Hepa 1-6 cells. After 24-hour incubation, cells were extracted by NP40 lysis buffer. Proteins were then electrophoresed using 10% and 12.5% polyacrylamide gel and transferred to PVDF membranes, which were closed with QuickBlock™ Closure Buffer and incubated with primary antibodies overnight at 4°C. Primary antibodies GPX4, SLC7A11, Frataxin, HSP70, HSP90, and β-actin were used for incubation. The PVDF membrane was washed three times with TBST before incubating the secondary antibody goat anti-rabbit IgG H & L (Alexa Fluor® 680) for 1 hour and visualizing the proteins.

*In vitro* Immunofluorescence staining

Tumor samples were isolated and collected from mice, which were subject to immunofluorescence staining including HSP70, DHE, and TUNEL tissue. Slides were washed with PBS and cell nuclei were stained with DAPI before sealing the slides. A microscope slide scanner was used for observation and analysis.

*In vivo* tumor protein blot analysis

Western blot method was used to detect the changes of glutathione GPX4, Frataxin, SLC7A11/Xct (R), HSP70 and HSP90 protein expression in tumors of nude mice at the end of treatment were isolated. The tumors protein was extracted by NP40 lysis buffer. Proteins were electrophoresed using 10% and 12.5% polyacrylamide gel and transferred to PVDF membranes, which were blocked with QuickBlock™ blocking buffer and incubated with primary antibodies overnight at 4°C. Primary antibodies GPX 4, SLC7A11, Frataxin, HSP70, and HSP90 were used for incubation. The PVDF membrane was washed three times with TBST before incubating the secondary antibody goat anti-rabbit IgG H & L (Alexa Fluor® 680) for 1 hour and visualizing the proteins.

*In vivo* safety evaluation

Four-week-old male nude mice were randomly divided into 2 groups (n = 3) and set up in two groups: G1 (Contorl) and G2 (DDP/MOF-Fc@HA+NIR). After day 18, blood was collected from the eyeballs of mice, followed by liver and renal function tests (aspartate aminotransferase, alanine aminotransferase, lactate dehydrogenase, creatinine, urea, and creatine kinase).

Haemolysis

To assess haemolysis *in vivo,* 1 mL of fresh blood was obtained from nude mice (male, 4 weeks old) by eyelid blood collection and centrifuged at 10,000 g for 5 mins to collect red blood cells (RBCs). 0.5 mL of RBC suspension was mixed in different concentrations of MOF-Fc@HA and DDP/MOF-Fc@HA at different concentrations respectively. The positive control: 0.5 mL of RBC suspension was mixed with 0.5 mL of distilled water. The negative control: 0.5 mL of RBC suspension was mixed with 0.50 mL of saline. 0.1 mL mixture was taken in a 96-well plate, and the absorbance at 450 nm was measured by an enzyme marker. The rest of the mixture was taken in a 0.9 mL EP tube and incubated at 37°C for 2 hours and photographed together. ** p* < 0.05, *** p* < 0.01, **** p* < 0.001.

Part B: Supplementary table and figures


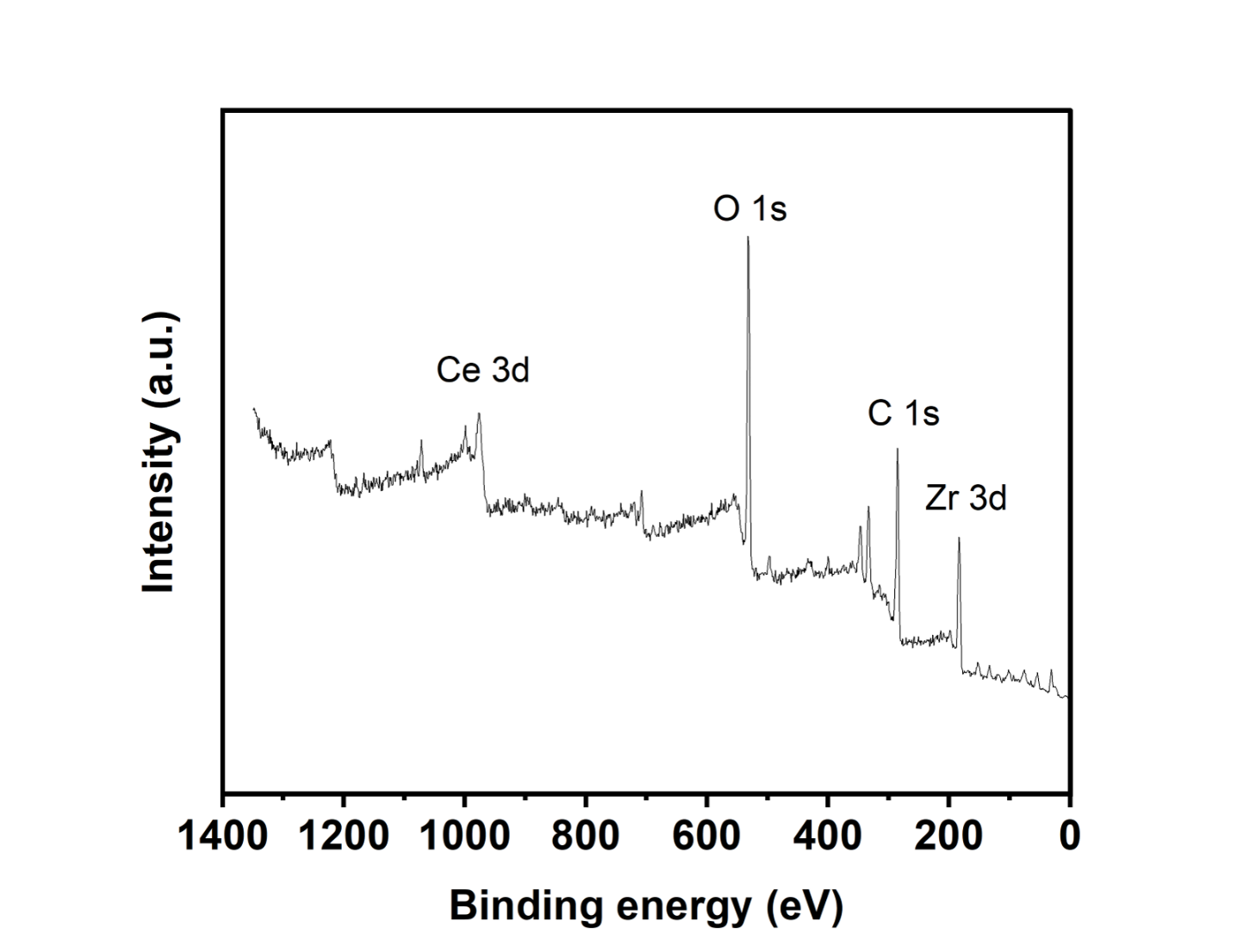


**Fig. S1.** X-ray Photoelectron Spectroscopy (XPS) of DDP/MOF-Fc@HA.


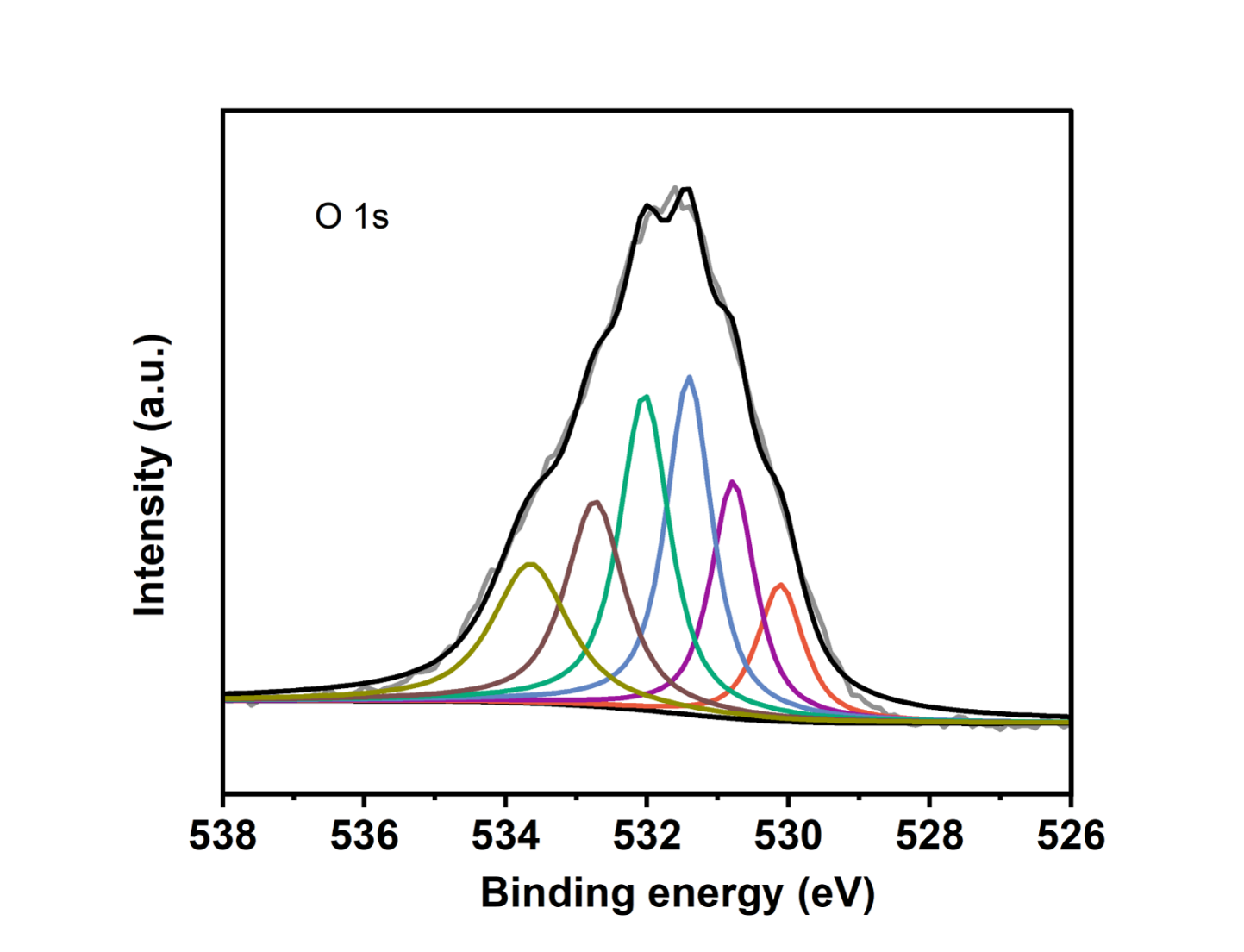


**Fig. S2.** The XPS of O 1s of DDP/MOF-Fc@HA.


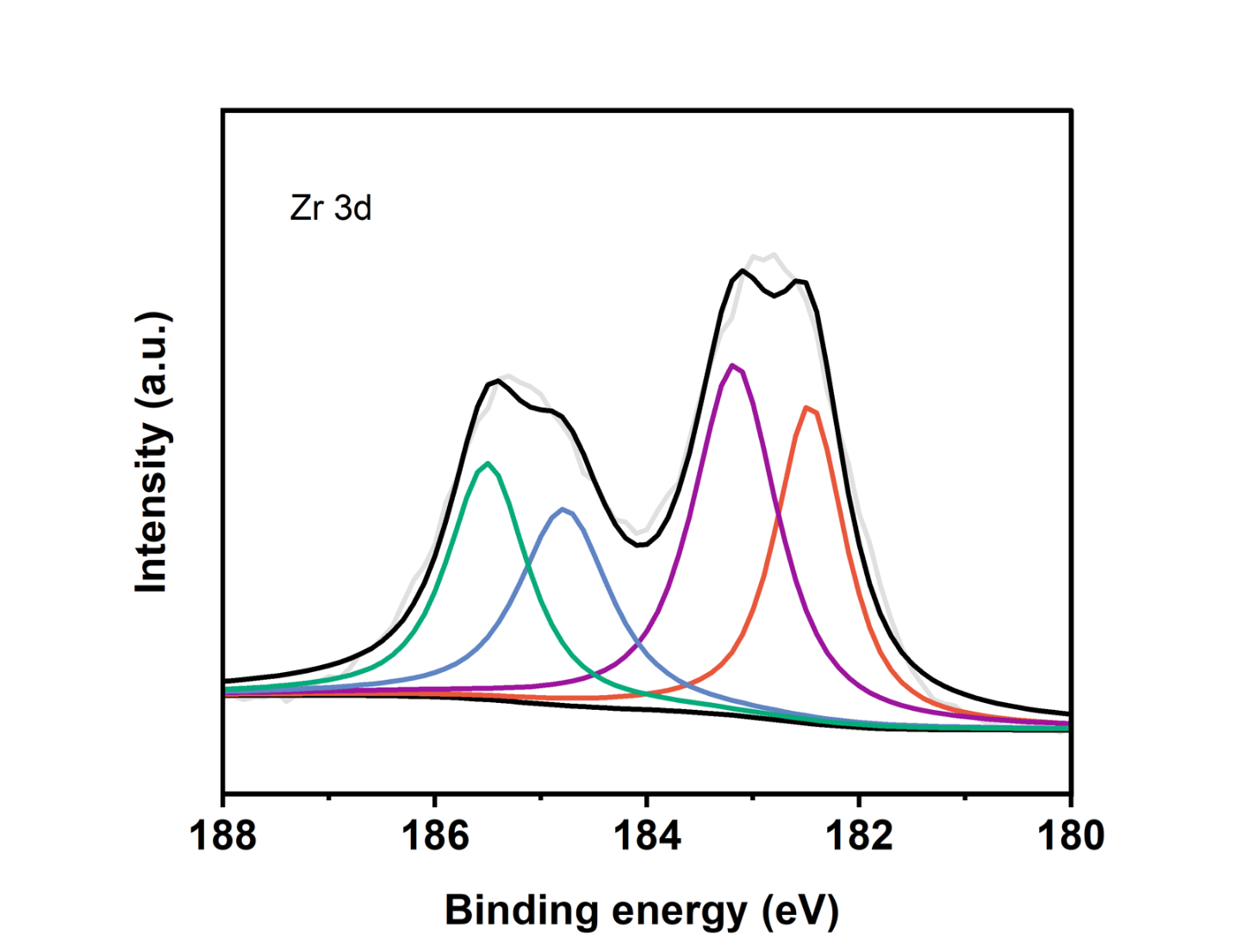


**Fig. S3.** The XPS of Zr 3d of DDP/MOF-Fc@HA.


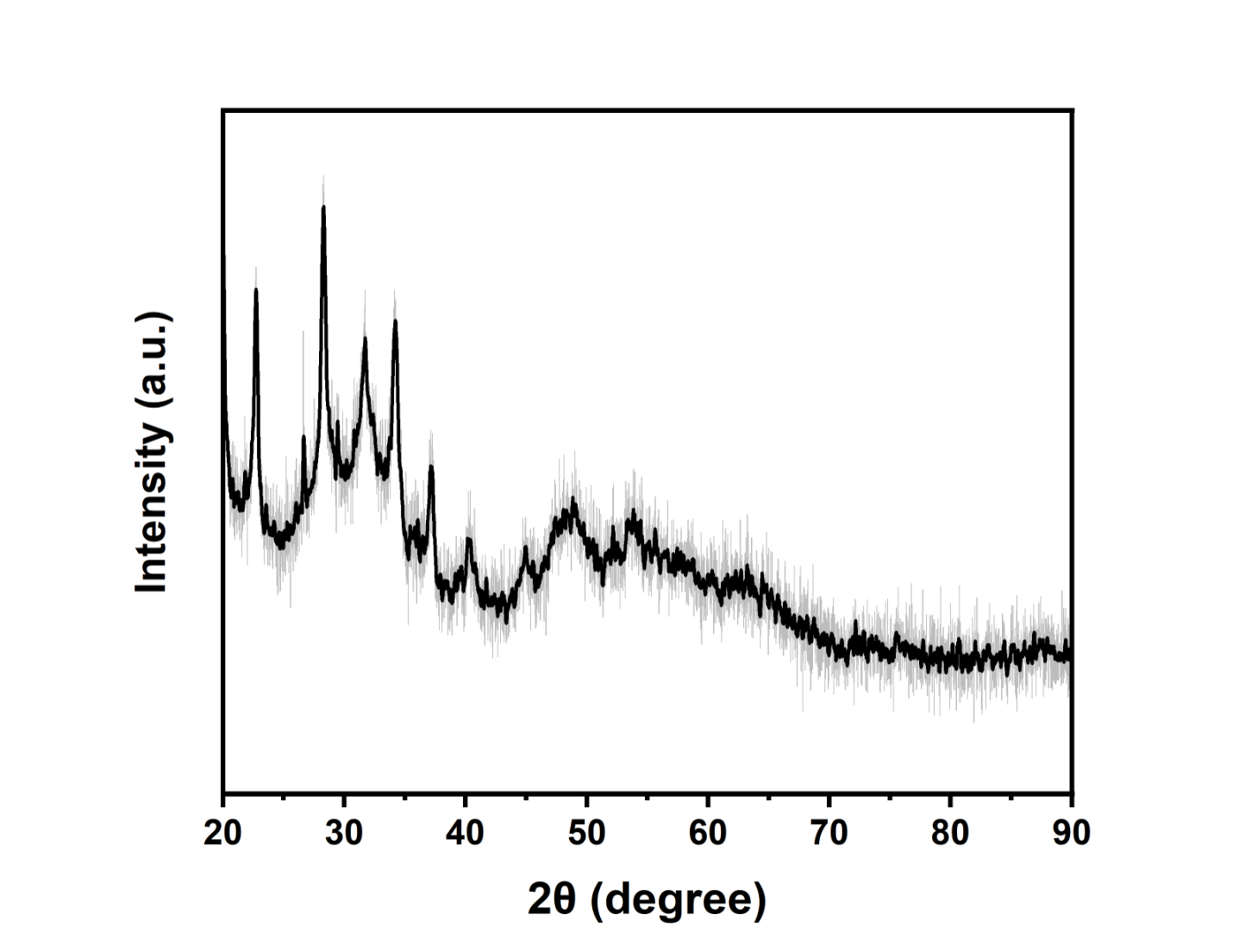


**Fig. S4.** X-Ray Diffraction (XRD) of MOF-Fc and DDP/MOF-Fc@ HA.


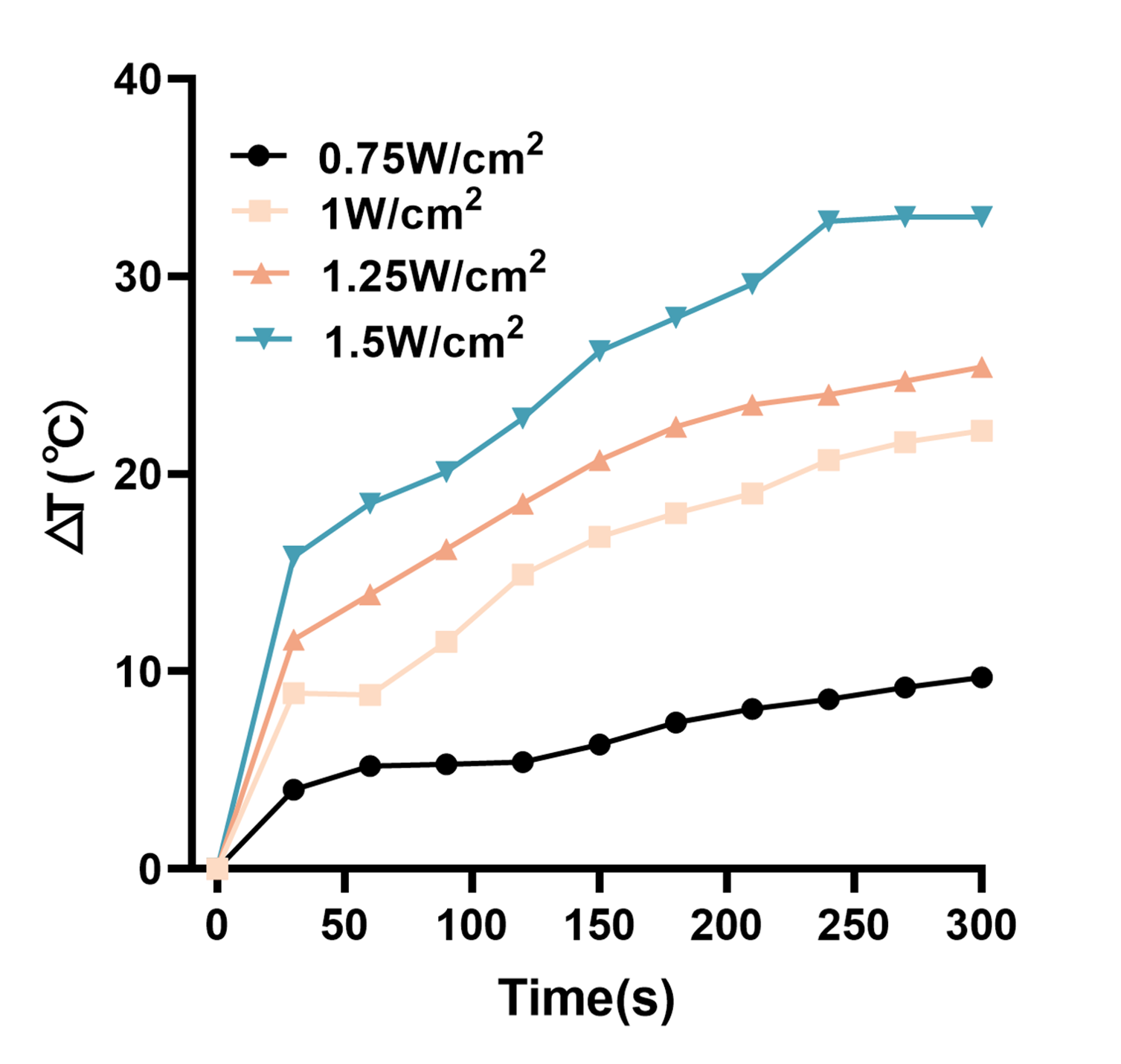


**Fig. S5.** Temperature variation curves of 100 μg mL^-1^ DDP@MOF-Fc aqueous solution with different laser powers (0.75, 1, 1.25, and 1.5 W cm^-2^) under 808 nm laser.


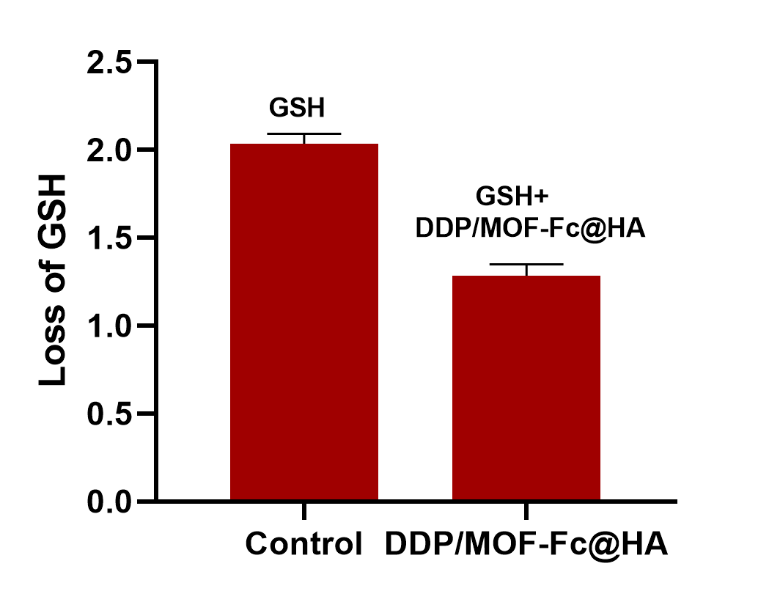


**Fig. S6.** Consumption of GSH with DDP/MOF-Fc@HA incubates in a water bath at 37 ◦C for 40 min.


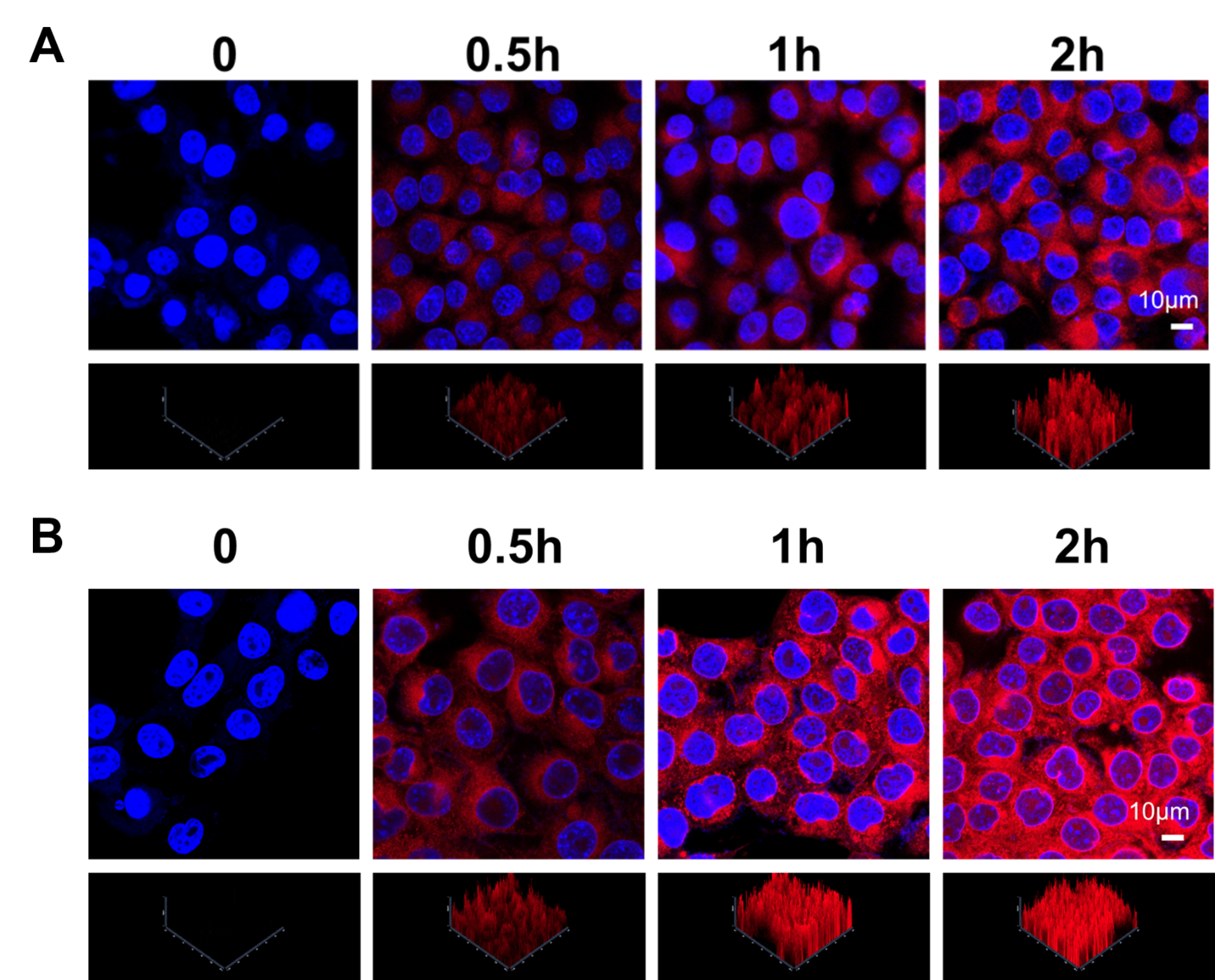


**Fig. S7.** (A) CLSM images of Hepa 1-6 and (B) 4T1 cells after incubation with IR780/DDP/MOF-Fc@HA NSs for different durations respectively (Red: IR780/DDP/MOF-Fc@HA NSs; Blue: DAPI) and the corresponding red fluorescence channel 2.5D plots.


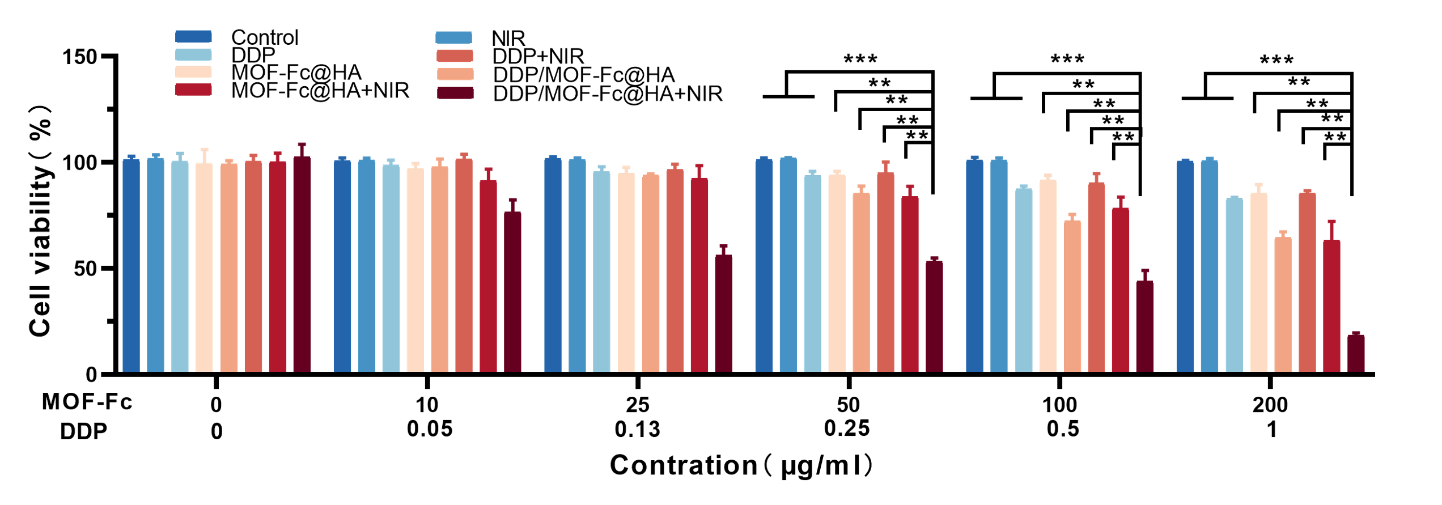


**Fig. S8.** The cell viability profiles of 4T1 cells after incubation with different concentrations of drugs under different treatments (n = 4).


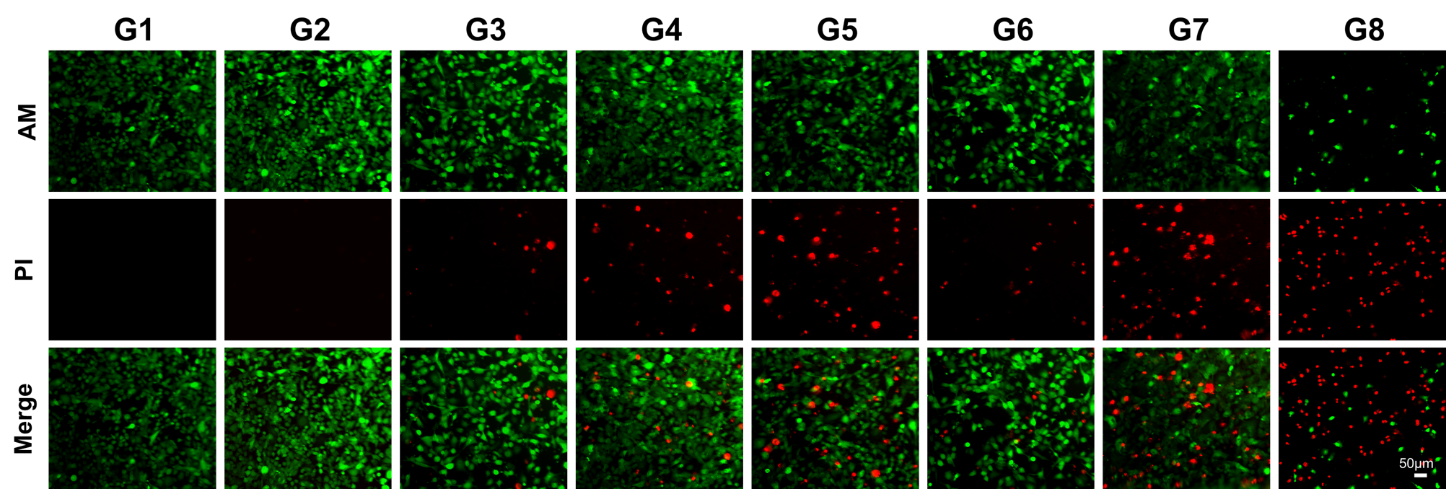


**Fig. S9.** 2D CLSM images of Hepa 1-6 cells after different treatments, performed after Calcein (AM)/PI co-staining.


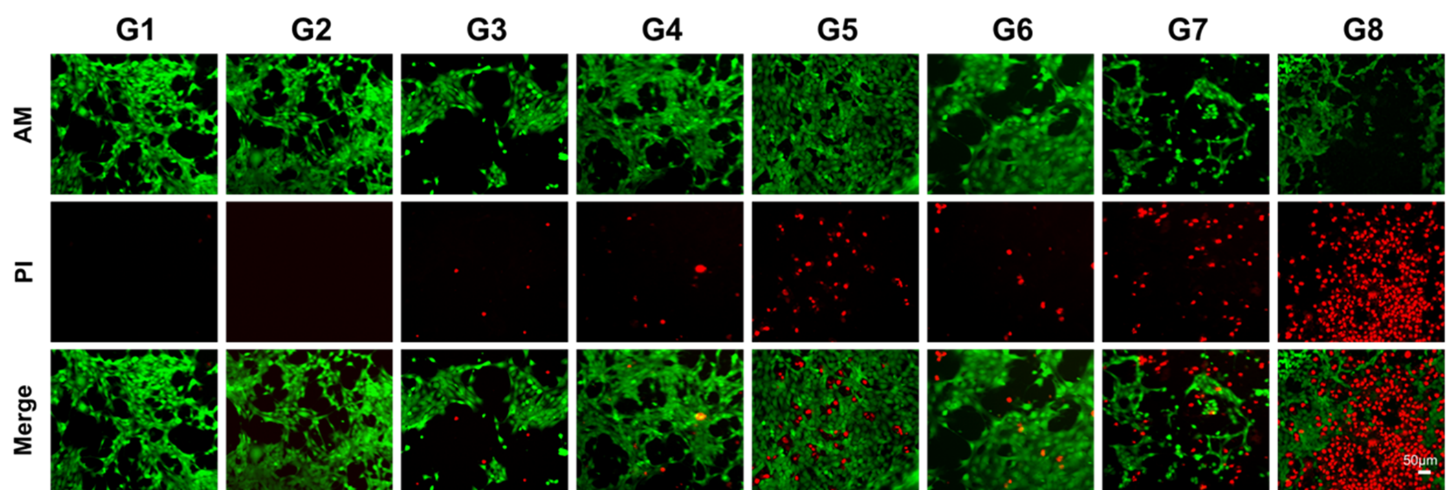


**Fig S10.** 2D CLSM images of 4T1 cells after different treatments, performed after Calcein (AM)/PI co-staining.


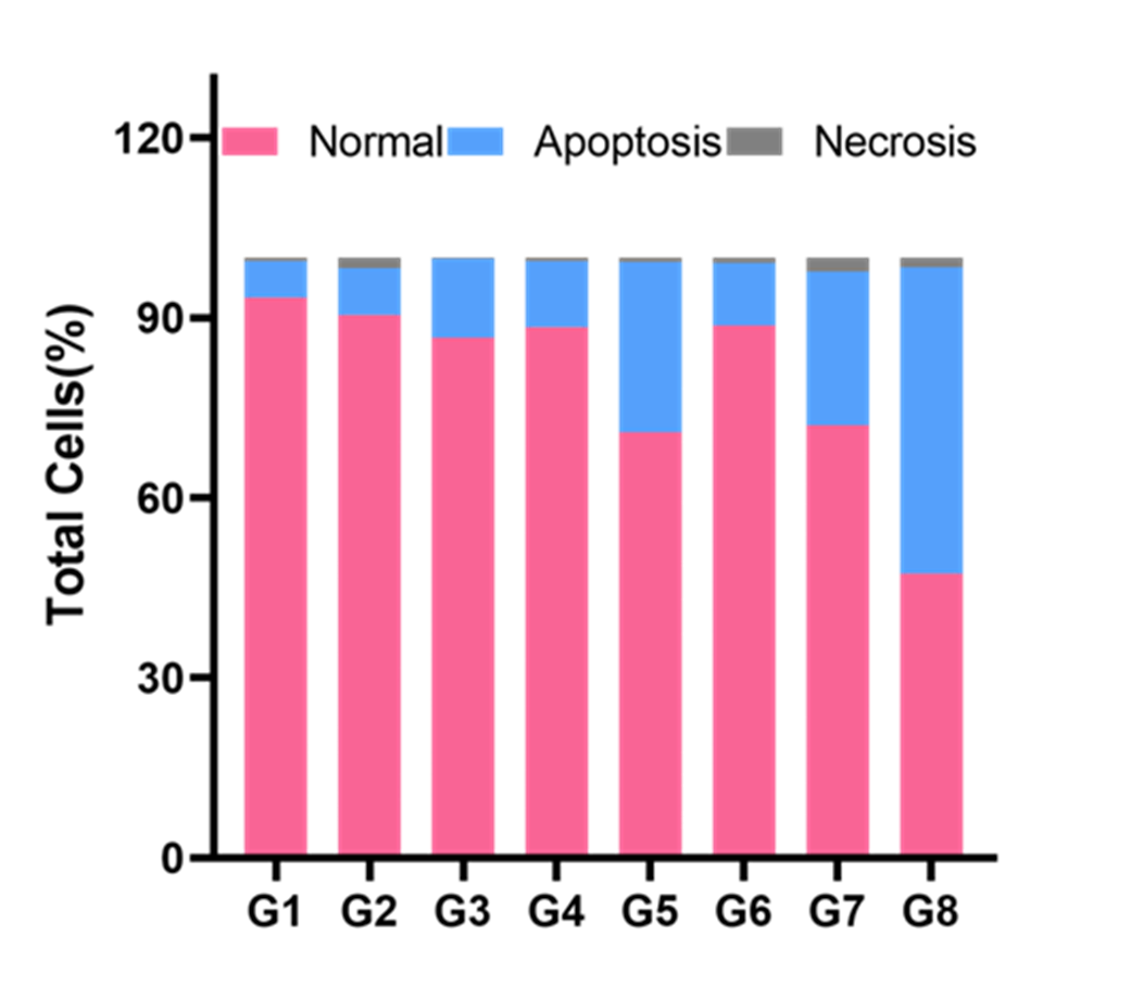


**Fig. S11.** Stacked histogram analysis was done for Hepa 1-6 cells after different treatments and after co-staining of Annexin V FITC/PI (Fig. 2g).


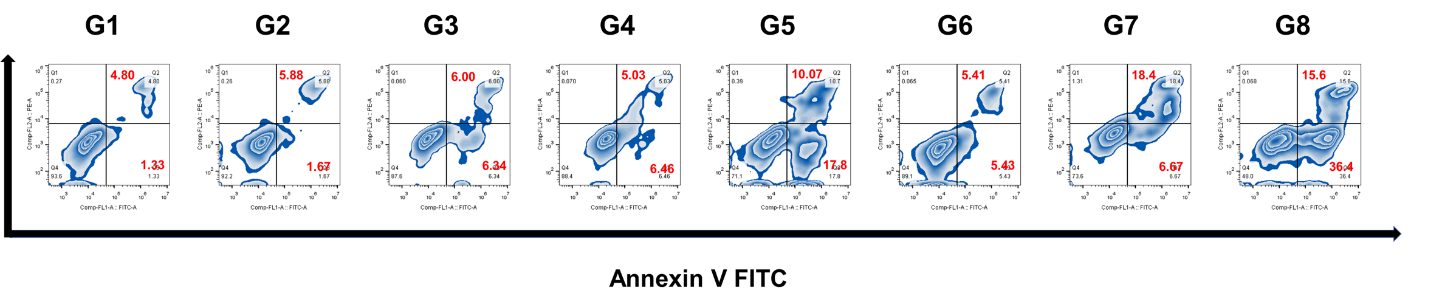


**Fig. S12.** Flow cytometry patterns of 4T1 cells cells after Annexin V FITC/PI co-staining.


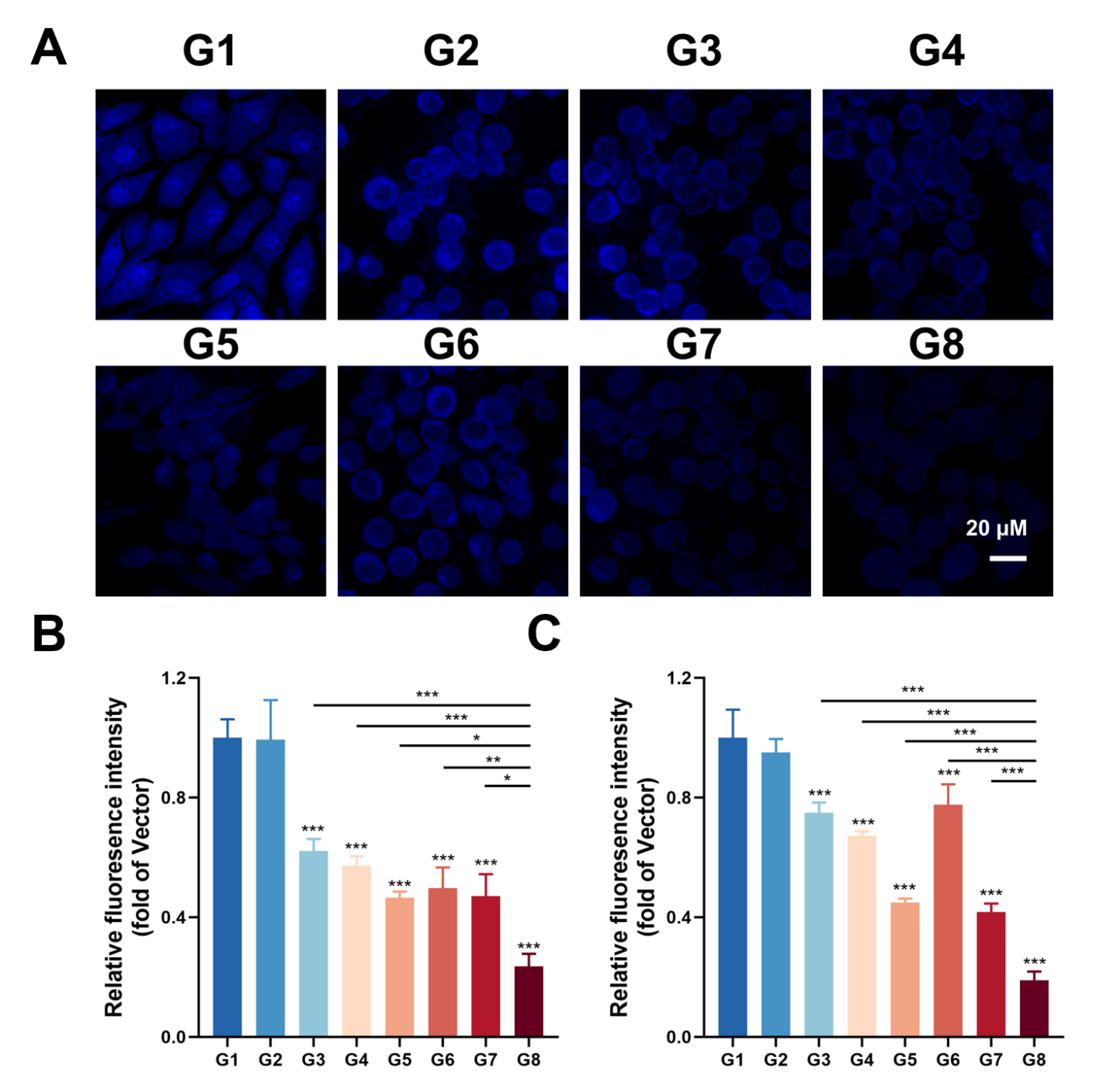


**Fig. S13.** (A) CLSM images of 4T1 cells after GSH indicator (i.e., ThiolTracker) after different treatments and (C) Corresponding quantitative fluorescence signal intensities. (B) Corresponding quantitative fluorescence signal intensities of CLSM images of Hepa 1-6 cells after staining with GSH indicator (i.e., ThiolTracker) after different treatments. * *p* < 0.05, ** *p* < 0.01, *** *p* < 0.001.


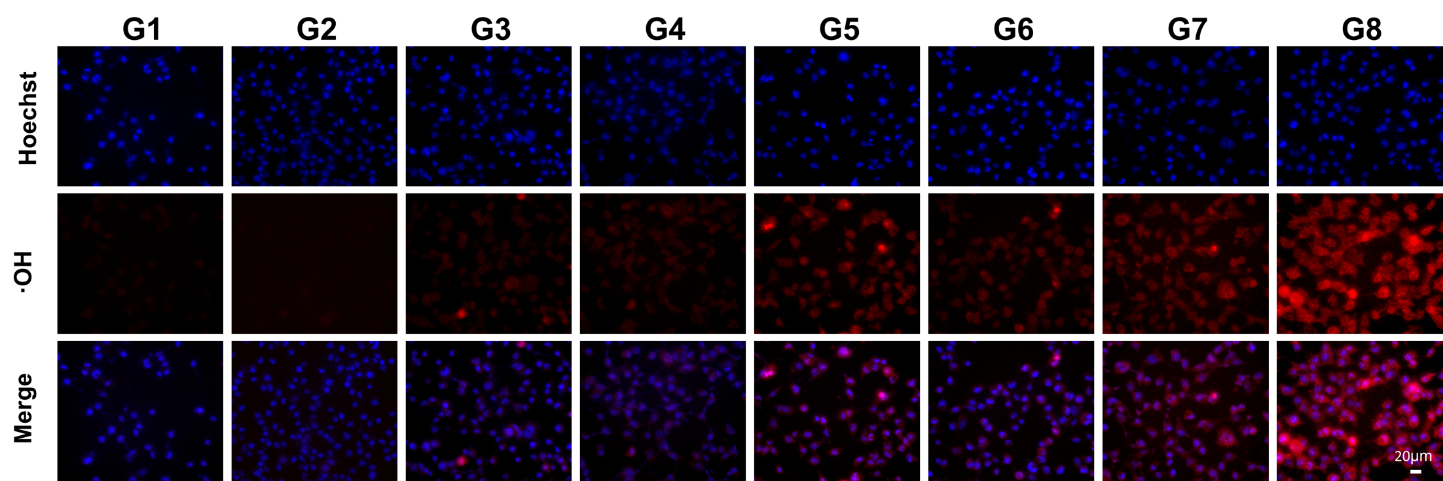


**Fig. S14.** CLSM images of Hepa 1-6 cells after with •OH indicator (i.e., hydroxyl radical fluorescent probe O28) after different treatments.


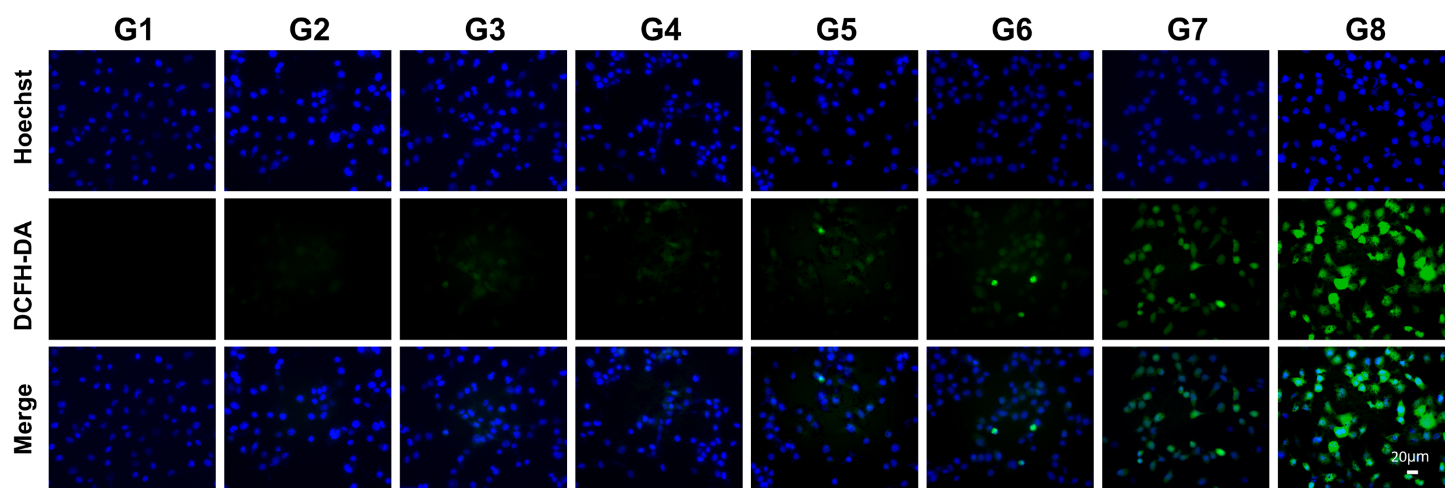


**Fig. S15.** CLSM images of Hepa 1-6 cells after after staining with ROS indicator (i.e., DCFH-DA) after different treatments.


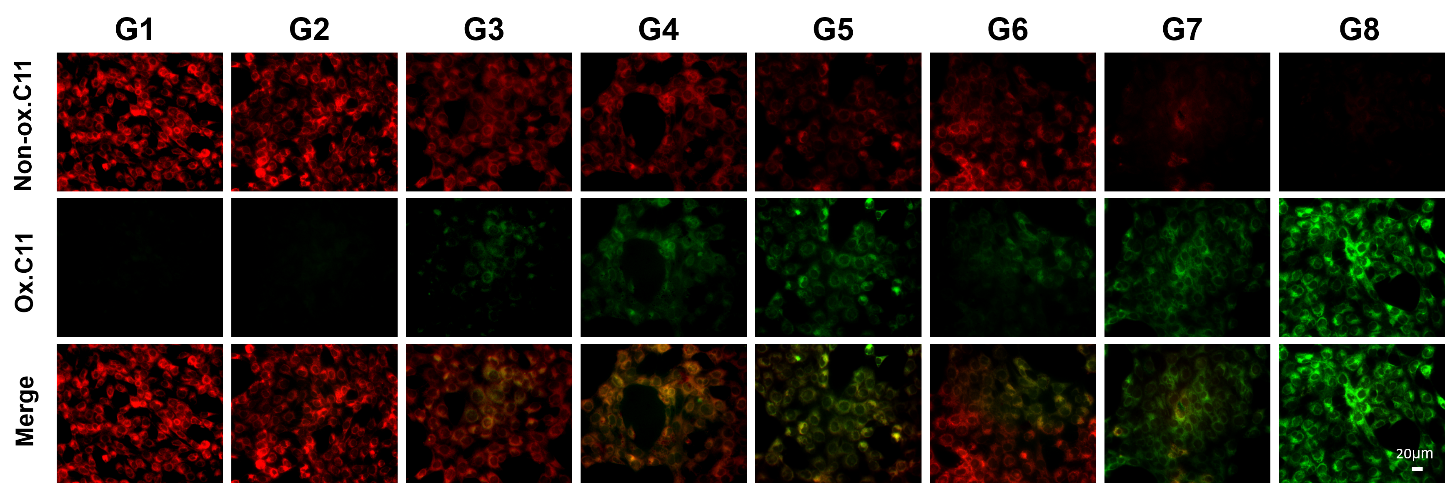


**Fig. S16.** CLSM images of 4T1 cells after Image-iT™ (lipid peroxidation).


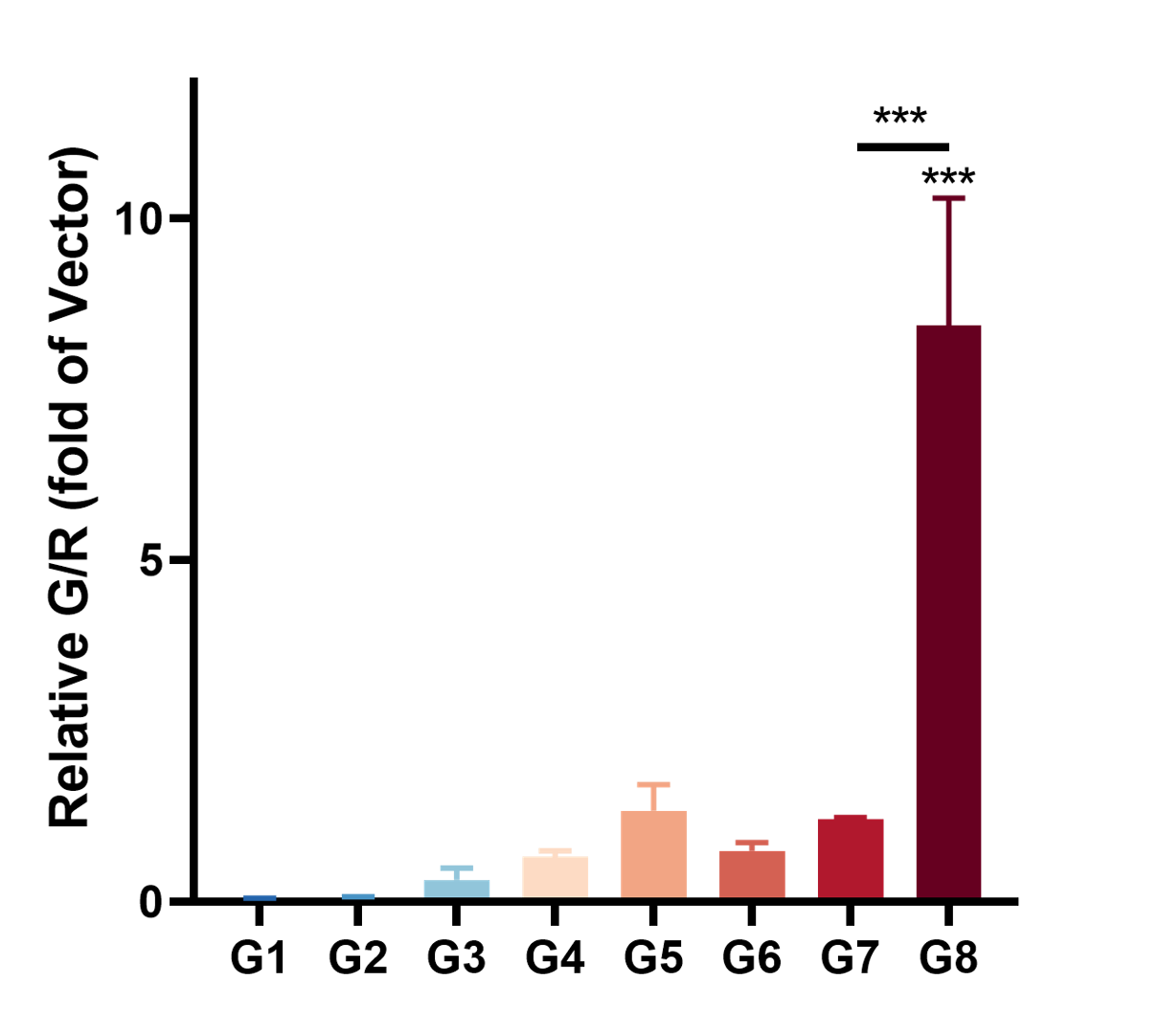


**Fig. S17.** The green to red of intensity fluorescence (G/R) ratio of CLSM images of Hepa 1-6 cells after JC-1 probe staining after different treatments. * *p* < 0.05, ** *p* < 0.01, *** *p* < 0.001.


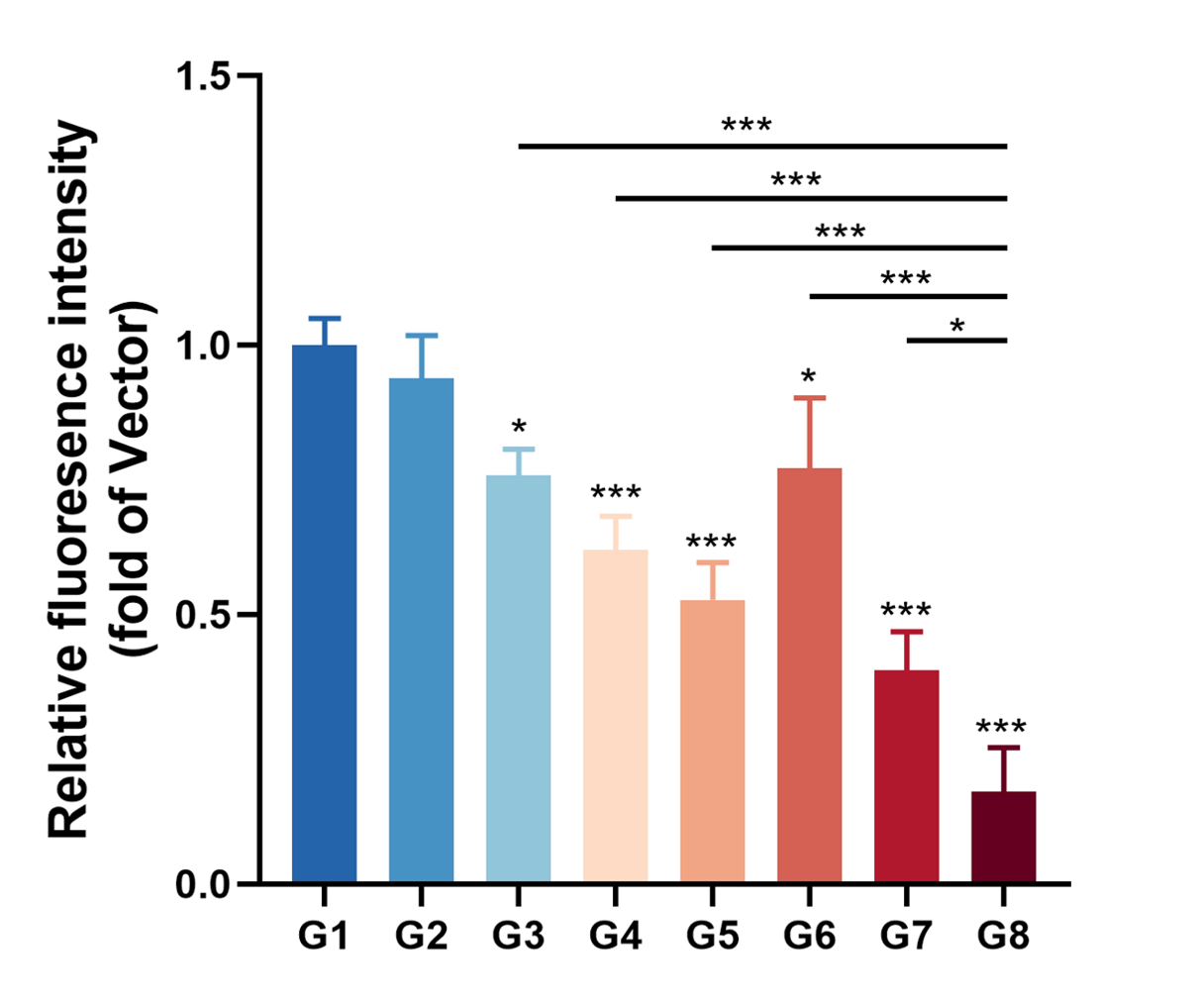


**Fig. S18.** Corresponding quantitative fluorescence signal intensities of CLSM images of Hepa 1-6 cells after staining with mitochondria probe staining (i.e., ThiolTracker) after different treatments (n=3).


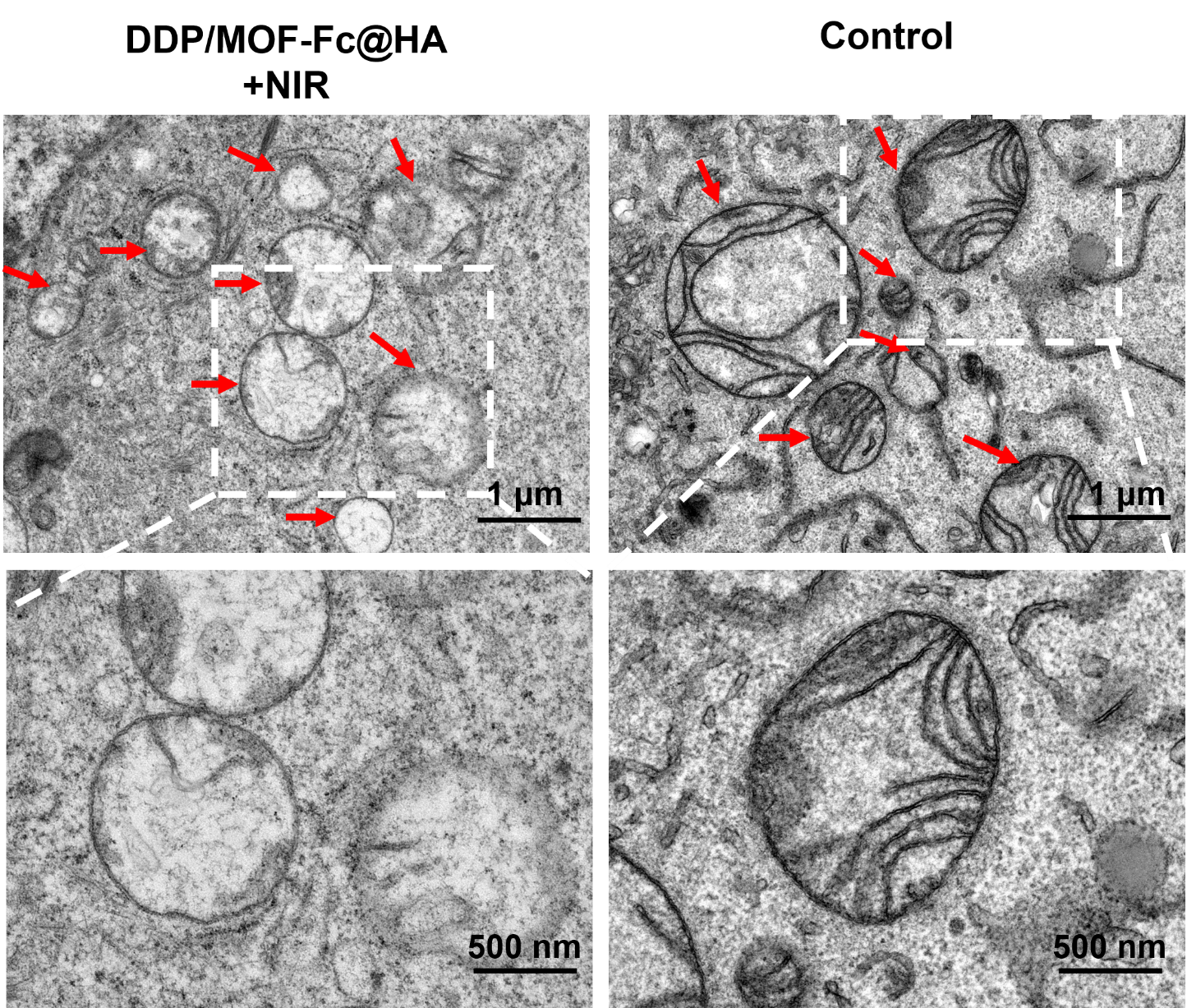


**Fig. S19.** Bio-TEM images of Hepa 1-6 cells after treatment with DDP/MOF-Fc@HA+NIR and the Control group respectively. (Scale bars: 1 μm and 500 nm).


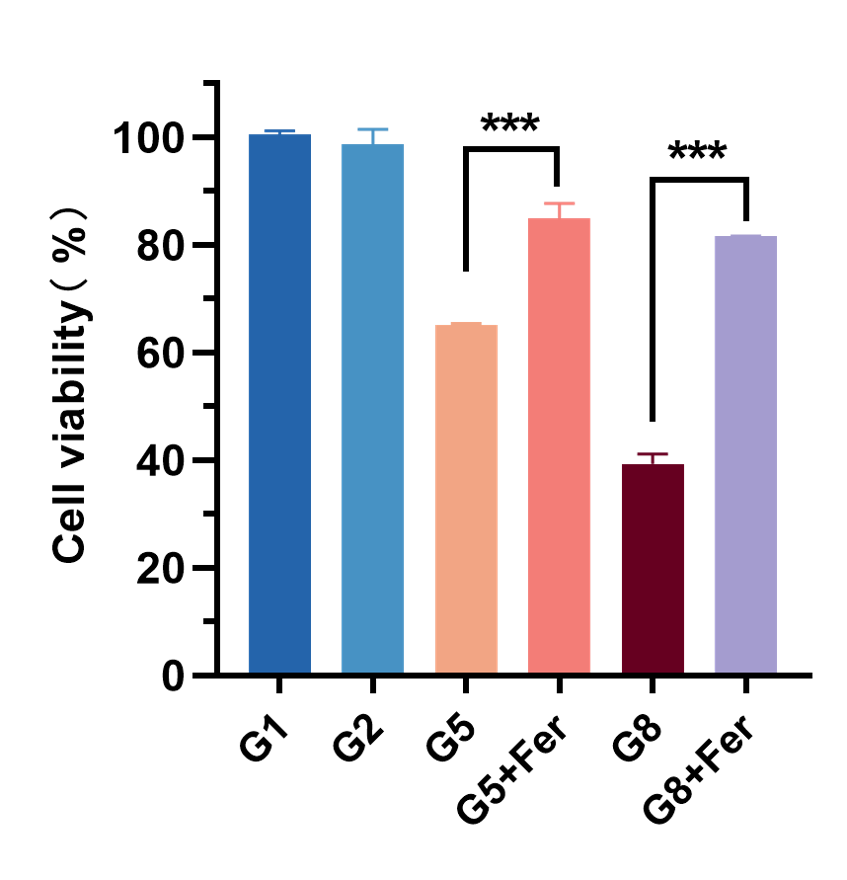


**Fig. S20.** The cell viability profiles of 4T1 cells after introduction ferrostatin-1 (Fer) (n = 4). * *p* < 0.05, ** *p* < 0.01, *** *p* < 0.001.


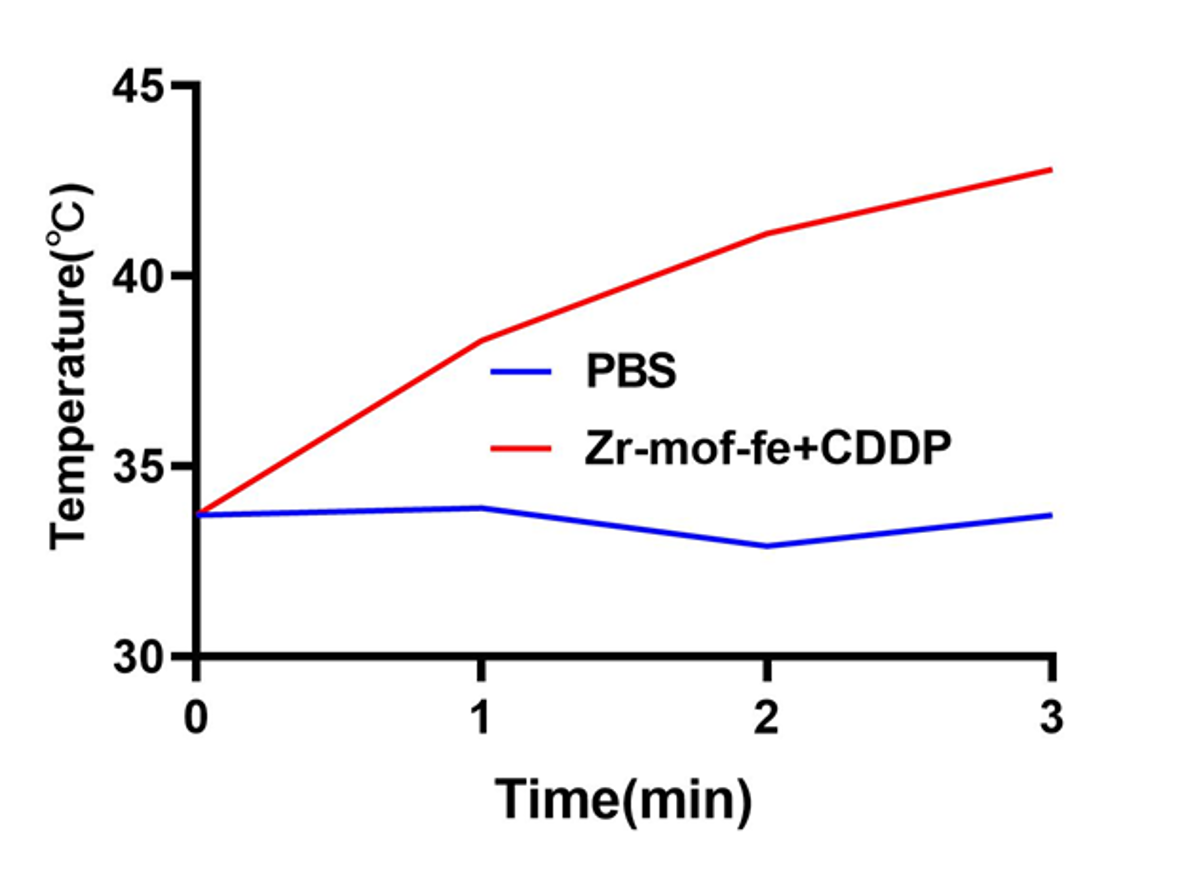


**Fig. S21.** Curve of elevated body temperature in mice when irradiating a tumor site for a specified length of time with NIR (808 nm, 1.5W cm^-2^, about 10 cm from the tumor surface) for 3 mins.


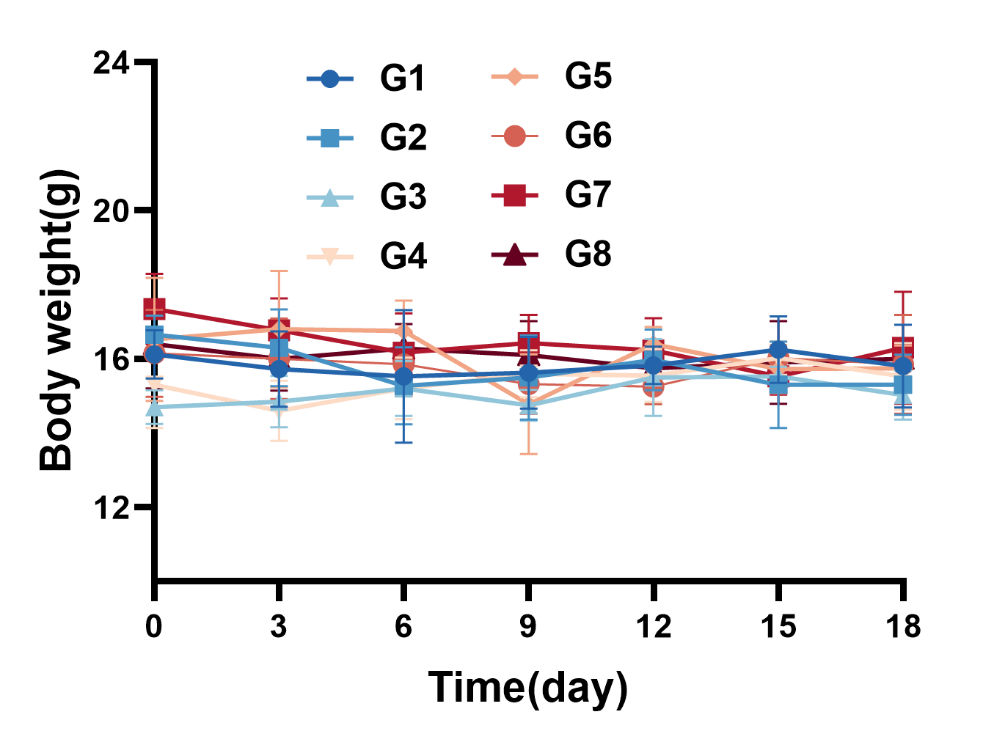


**Fig. S22.** Changes in body weight of mice in each group during the experiment. (n = 4). * *p* < 0.05, ** *p* < 0.01, *** *p* < 0.001.


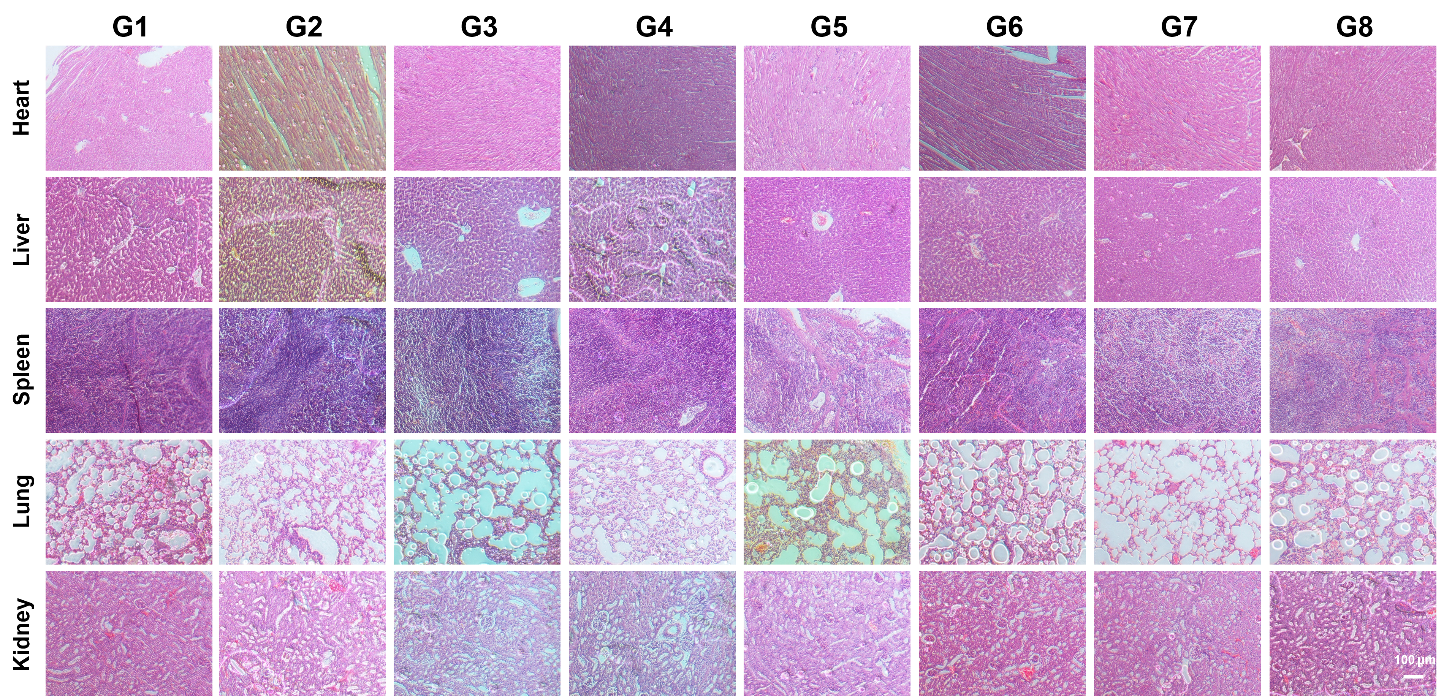


**Fig. S23.** H&E light microscopy images of organ sections isolated from mice of different treatment groups at the end of the experimental period.


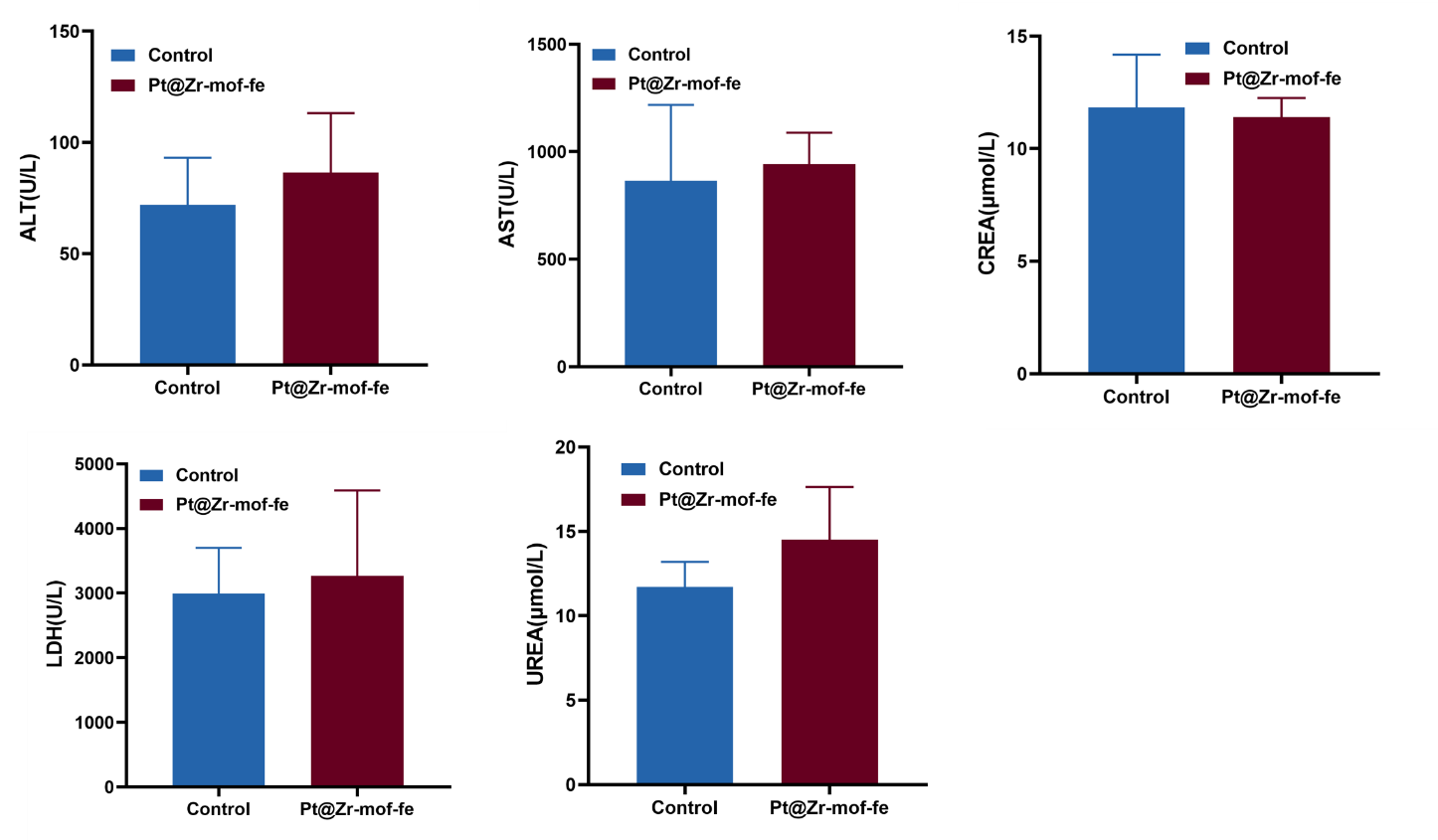


**Fig. S24.** Quantitative data on haematological and biochemical indices of control mice and final group of treated mice. Data are expressed as mean ± SD (n = 3). * *p* < 0.05, ** *p* < 0.01, *** *p* < 0.001.


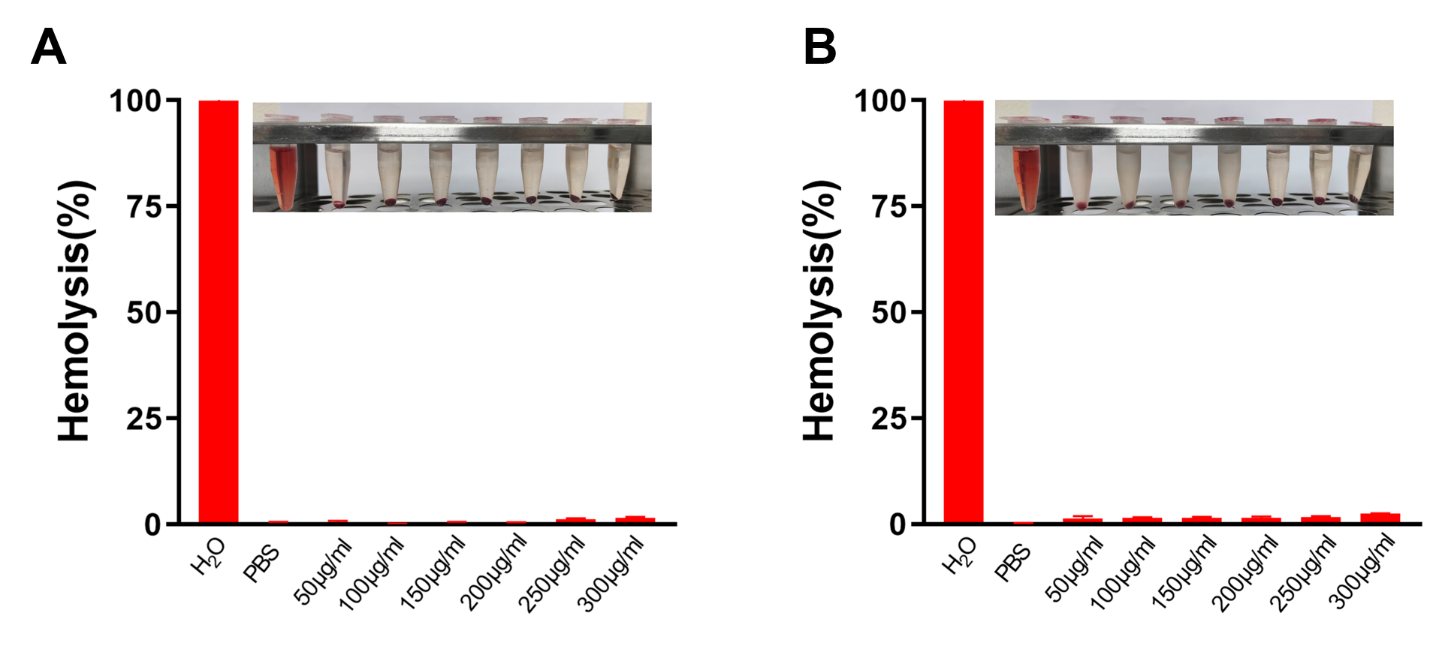


**Fig S25.** (A) MOF-Fc@HA and (B) DDP/MOF-Fc@HA at different concentrations haemolysis assay (n = 3). * *p* < 0.05, ** *p* < 0.01, *** *p* < 0.001.


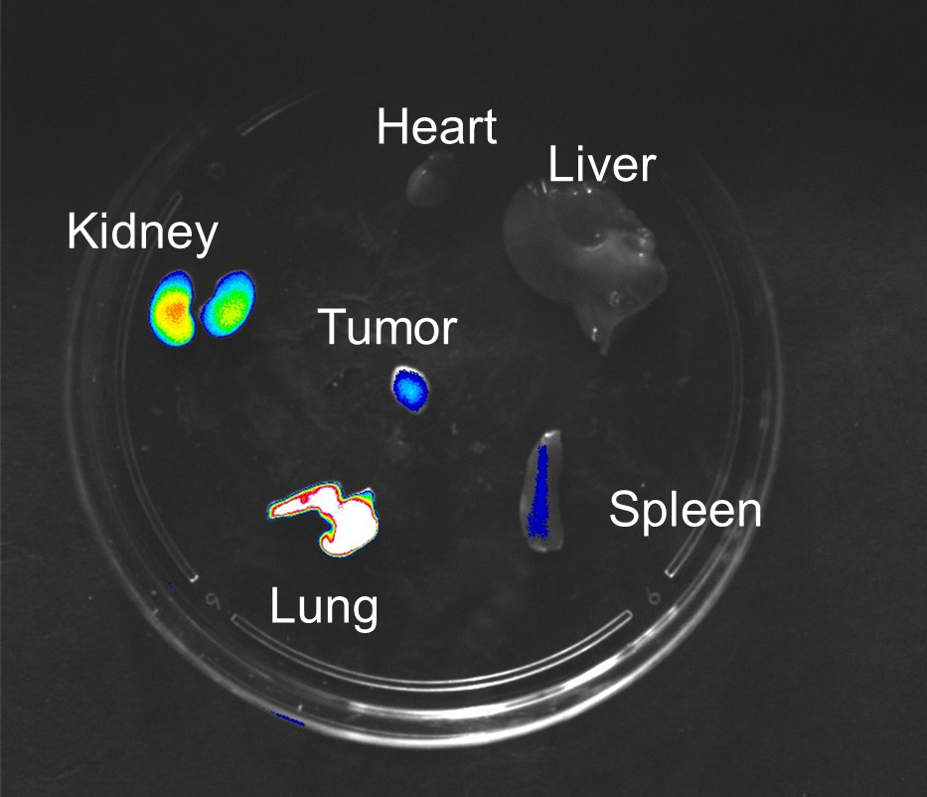


**Fig. S26.** Fluorescence images of isolated nice tumors and organs 24 hours later.

| Condition | K_m_ _[mM]_ | υ_max_ _[10_^-8^ _M S_^-1^_]_ |
| --- | --- | --- |
| 25 ℃ | 6.02 | 10.25 |
| 37 ℃ | 6.15 | 16.37 |
| 43 ℃ | 7.17 | 35.21 |

**Table S1.** Kinetic parameters of the enzyme DDP/MOF-Fc@HA, including Mie constant (K_m_) and maximum reaction rate (υ_max_).
